# Outcomes of selective dorsal rhizotomy in ambulatory children and young people with cerebral palsy: A scoping review

**DOI:** 10.1111/dmcn.16496

**Published:** 2025-09-19

**Authors:** Deepti Chugh, Eleanor Main, Gillian Waite, Lucy Alderson, Kristian Aquilina, Cherry Kilbride, Tim Theologis, Hortensia Gimeno

**Affiliations:** ^1^ Physiotherapy, Infection, Immunity, Inflammation Department UCL Great Ormond Street Institute of Child Health, UCL London UK; ^2^ Physiotherapy, Great Ormond Street Hospital for Children NHS Foundation Trust London UK; ^3^ Department of Neurosurgery Great Ormond Street Hospital for Children NHS Foundation Trust London UK; ^4^ College of Health, Medical and Life Sciences, Brunel University of London UK; ^5^ Therapy Services Royal Free London NHS Foundation Trust London UK; ^6^ Nuffield Department of Orthopaedics, Rheumatology and Musculoskeletal Sciences University of Oxford, Botnar Research Centre Oxford UK; ^7^ Barts Bone and Joint Health, Blizard Institute Queen Mary University of London London UK; ^8^ The Royal London Hospital and Tower Hamlets Community Children's Therapy Services Barts Health NHS Trust London UK

## Abstract

**Aim:**

To identify outcomes reported after selective dorsal rhizotomy (SDR) in ambulant children and young people with cerebral palsy in different domains of the International Classification of Functioning, Disability and Health (ICF).

**Method:**

A scoping review using the JBI Scoping Review methodology was conducted. Six databases were searched for literature published between 1993 and 2024.

**Results:**

A total of 214 published papers met the inclusion criteria. Outcomes under the body function and structure domain were most frequently investigated (*n* = 199, 93%), followed by activity (*n* = 123, 58%) and participation (*n* = 33, 15%) across all studies. Quality of life was reported in 16 (8%) studies, and four (2%) studies mentioned individualized goals for SDR surgery. A combination of validated measures and subjective outcomes was used, with 119 (56%) studies reporting outcomes in two or more domains.

**Interpretation:**

Impairment‐based outcomes remain the primary focus in SDR research. A small shift in emphasis towards participant‐reported outcome measures has been seen in recent years. Few studies reported on the impact of personal and environmental factors. Future SDR studies need to incorporate all domains of the ICF to enhance understanding and capture holistic, meaningful changes in the lives of children and young people with cerebral palsy and their families.

## Graphical abstract

This scoping review highlights that in the selective dorsal rhizotomy (SDR) literature, the emphasis still remains on impairment‐based outcomes in the body function and structure domain of the ICF. However, there has been a small shift towards participation and quality‐of‐life outcomes in the last decade. While many studies focus on the quantitative outcome data of SDR, little research has explored children and young people's and parental perspectives around SDR.

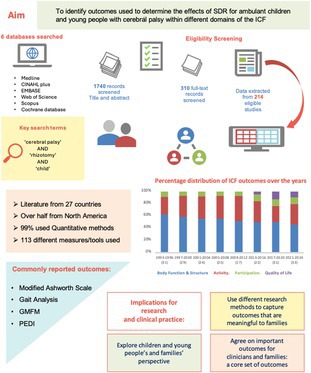

Plain language summary: https://onlinelibrary.wiley.com/doi/10.1111/dmcn.70048

**Abbreviations**
ICF: International Classification of Functioning, Disability and Health
SDR: selective dorsal rhizotomy

**What this paper adds**

- Impairment‐based outcomes continue to be the primary focus of research on selective dorsal rhizotomy (SDR).
- There is inconsistent reporting of contextual factors.
- Highlights the paucity of studies providing children and families perspectives.
- There is a need to include and report individual goals of SDR in clinical practice.
- Developing core clinical and research outcome sets will standardize reporting across the lifespan.

Selective dorsal rhizotomy (SDR) is an irreversible neurosurgical procedure which reduces spasticity in the lower limbs of children and young people with bilateral spastic cerebral palsy (CP).^1^, ^2^ Research suggests that SDR combined with intensive physiotherapy can improve gross motor function, activity, independence, participation, and quality of life.^3^, ^4^ However, systematic reviews^5^, ^6^ have found limited evidence on the longer‐term effectiveness of SDR on functional mobility, self‐care activities, and participation, potentially because of low‐level evidence and bias in the SDR literature. In contrast, some longitudinal observation studies have reported overall gain in gross motor function compared with the natural history of CP.^7^, ^8^

Despite the conflicting evidence, globally there is an increased recognition and uptake of SDR as a permanent surgical intervention for reducing spasticity. Given the irreversible nature of SDR and the uncertainty of longer‐term outcomes, decision‐making for families can be challenging.^9^ A significant knowledge gap remains in understanding which outcomes are most relevant and meaningful for children and families. Identifying the measures used at various stages of follow‐up is essential for clarifying the impact of SDR on children and young people with bilateral spastic CP and their families' lives.

The International Classification of Functioning, Disability and Health (ICF)^10^ framework has been used in systematic reviews to determine SDR effectiveness.^5^, ^6^ The multidimensionality of the SDR outcomes across the ICF lends to a flexible and comprehensive approach to the synthesis of diverse evidence. In this bio‐psycho‐social model, the impairments of an individual caused by disability are considered in the context of environmental and personal factors. Understanding the relationship between different domains of the ICF ‘body functions and structure’ (e.g. muscle tone, muscle weakness, joint mobility, deformities), ‘activity’ (e.g. execution of a task or action, mobility, self‐care), and ‘participation’ (e.g. involvement in a life situation, playing sports, engaging in leisure activities) and contextual factors such as external ‘environmental factors’ (social, physical, and legislative) and internal ‘personal factors’ (age, education, social background, motivation, psychological impact) is essential to identify problem areas, tailor management interventions to the individual's needs, and determine the effectiveness of interventions.^11^ The ICF provides a common language to describe an individual's health condition and functional abilities, facilitates communication between clinicians and families, allows data comparison across countries, and helps to promote a more holistic understanding of the health‐related outcomes and provision of family‐centred care.^12^

Healthcare delivery and reporting of outcomes have evolved to emphasize the broader impact of an intervention on quality of life and family experiences.^13^ The increasing use of the ICF framework in children and young people with bilateral spastic CP research has shifted the focus towards participation outcomes and the influence of environmental factors.^14^ Although various outcomes have been reported to be related to SDR in children and young people with bilateral spastic CP, it remains unclear which ICF domains are most prominent or whether reporting patterns or the choice of outcome measures have changed since the introduction of the ICF in 2001.

Considering that many studies are excluded from systematic reviews because of low‐quality evidence, this scoping review expands previous work by examining the full spectrum of outcomes reported for ambulatory children and young people with bilateral spastic CP, in all types of SDR study over the past 30 years—a period during which SDR became a mainstream treatment for lower‐limb spasticity. This review does not assess the effectiveness of SDR or the validity of the outcome measures. Instead, by mapping outcomes across ICF domains, it highlights research trends, identifies gaps, and informs selection of key outcomes for clinical practice and future SDR research on children and young people with bilateral spastic CP and their families. The scoping‐review research questions were the following. (1) What outcomes are reported in the literature for ambulatory children with bilateral spastic CP at different stages of follow‐up after SDR? (2) How do these outcomes map to the domains of body structure and function, activity, participation, and contextual factors in the ICF or in the context of quality of life? (3) Which measurement or evaluation tools are used after SDR to capture these outcomes?

## METHOD

A preliminary search of MEDLINE, the Cochrane Database of Systematic Reviews, JBI Evidence Synthesis, and Prospero^15^ was conducted; no current or ongoing systematic or scoping reviews on the topic were identified. The inclusion criteria for this scoping review were guided by the population, concepts, and context approach.^15^ The review protocol was registered in the Open Science Framework register (https://doi.org/10.17605/OSF.IO/FJXA6).^16^

### Participants

This review considered all studies that included ambulatory children and young people with a diagnosis of bilateral spastic CP (Gross Motor Function Classification System [GMFCS] levels I, II, and III) who had SDR surgery at some point before 18 years of age. Studies including children and young people with diagnoses other than CP or classified in GMFCS levels IV or V only were excluded.

### Concept

The concept of interest was all outcomes reported after SDR. Only studies reporting outcomes of SDR performed at the lumbosacral level were considered. Any other variants of the surgical procedure or surgical site (e.g. cervical spine) were excluded.

### Context

All SDR outcomes, including adverse events and complications, reported from 1993 to 2024, related to body structure and function, activity, participation, and contextual factors as defined by the ICF.^10^ Quality‐of‐life outcomes, which encompass all components of the ICF, patients' experience, individual goals, and satisfaction with the outcomes of the procedure, were also included.

### Types of evidence source

All study designs such as randomized controlled trials, non‐randomized controlled trials, before‐and‐after studies, prospective and retrospective cohort studies, case–control, case series, and case reports were included. Qualitative studies focusing on the outcomes of SDR were also included. Studies describing the surgical or electrophysiological procedure of SDR, cost‐effectiveness, or service delivery were excluded if no outcomes were reported. Abstracts, reviews, opinion papers, and commentaries were also excluded.

### Search strategy

An initial limited search of MEDLINE was undertaken to identify articles on the topic. The text words in the titles and abstracts of relevant articles and the index terms used to describe the articles were used to develop a full search strategy for MEDLINE using OVID platforms. The search strategy, including all identified keywords and index terms, was adapted for each included database. The search strategy was reviewed by a medical librarian according to the guidance in Peer Review of Electronic Search Strategy (PRESS).^17^ No restrictions were placed on the publication date or language for the initial search.

### Selection of sources of evidence

Databases searched include Medline and CINAHL Plus with Ovid, Embase with EBSCOhost, Web of Science, Scopus, and Cochrane Database of Systematic Reviews. The key search terms included ‘cerebral palsy’ AND ‘rhizotomy’ AND ‘child’. Detailed search terms are included in Appendix S1 for all six databases.

### Data extraction and charting

A spreadsheet was developed and piloted to gather information about study type, location, GMFCS level, length of follow‐up and outcome domains, measures or tools used, and adverse events and complications (Appendix S2). Data extraction was completed by two reviewers (DC and GW). The level of evidence was reported using the Oxford Centre for Evidence‐based Medicine Scale,^18^ which was predominantly used to categorize studies on the basis of the study design. Critical appraisal of studies was not conducted as part of this review.

### Collation and summarizing results

Outcomes were mapped into different domains of ICF body structure and function, activity, participation, and contextual factors on the basis of the ICF definitions.^10^ Quality‐of‐life outcomes, which encompass all components of the ICF, patient experience, individual goals, and patient satisfaction, were categorized separately. Any uncertainty about the categorization of the outcomes was discussed among four researchers (DC, GW, LA, HG), and a consensus reached on the basis of the previous literature and published content validity of the outcome measures. Outcome measures that captured more than one domain of the ICF, such as the Pediatric Evaluation of Disability Inventory^19^ and Wee Functional Independence Measure,^20^ were included in both the activity and participation domains of the ICF. Similarly, most survey studies captured outcomes across all domains of the ICF and were mapped accordingly. Descriptive analysis, including counts, percentages, and synthesis, was performed in Microsoft Excel version 16.94.

### Study selection

The search was performed in May 2022 and updated in July 2024. The primary reviewer (DC) ran the initial searches and uploaded all citations into EndNote X9.3.3/2020 (Clarivate Analytics, PA, USA). After removing duplicates, all citations were imported into the Rayyan literature review tool (Rayyan Systems, Cambridge, MA, USA).^21^ Titles and abstracts were screened by two independent reviewers (DC, GW) for eligibility against the inclusion criteria. The full text of potentially relevant studies (*n* = 310) was assessed in detail against the inclusion criteria by two independent reviewers (DC and GW), who were blinded to each other's screening decisions. Reasons for excluding studies that did not meet the inclusion criteria were recorded in the Rayyan online software and are provided in Table S1. Any disagreements between the reviewers at each stage of the selection process were resolved through discussion or with an additional reviewer (HG). The search results and study inclusion process are presented in a Preferred Reporting Items for Systematic Reviews and Meta‐analyses extension for scoping review (PRISMA‐ScR) flow diagram^22^ (Figure S1).

## RESULTS

### Characteristics of studies included

This review included 214 studies (Table S2). Seven studies reported outcomes using randomized controlled trial designs.^23^, ^24^, ^25^, ^26^, ^27^, ^28^, ^29^ Longitudinal observational studies were the most common study design (*n* = 94, 44%) and retrospective cohort studies (*n* = 91, 43%), followed by surveys (*n* = 12, 6%),^30^, ^31^, ^32^, ^33^, ^34^, ^35^, ^36^, ^37^, ^38^, ^39^, ^40^, ^41^ case reports (*n* = 6, 3%),^42^, ^43^, ^44^, ^45^, ^46^, ^47^ and two qualitative studies^48^, ^49^ reporting parental and children's experiences after SDR. The level of evidence of studies was primarily graded as level IV (*n* = 178, observational studies), followed by level III (*n* = 21, with a control group), level V (*n* = 6, case reports), and level II (*n* = 7, randomized controlled trials). Most studies were conducted in North America (50%), European countries (24%), the Western Pacific region (18%), and Africa (6%) (Appendix S3). Sample sizes ranged from 1 to 785 (median 33, interquartile range 19–75). Participants' ages typically ranged from 3 years to 28 years. All study and participants' characteristics are presented in Table 1. While the primary focus was on ambulant children and young people with bilateral spastic CP, classified in GMFCS levels I to III, several studies also included participants in GMFCS levels IV and V. The GMFCS levels and topological classifications presented in Table 1 reflect the overall composition of the study samples.

**TABLE 1:** Characteristics of included studies (*n* = 214) and participants' characteristics (*n* = 13 530).

Characteristics of included studies (*n* = 214)			
Study characteristics		*n*	%
Year of publication			
	1993–2002	73	34.1
	2003–2012	55	25.7
	2013–2024	86	40.2
World Health Organization regions			
	North America	107	50
	Europe	52	24.3
	Western Pacific	38	17.8
	Africa	12	5.6
	South America	3	1.4
	Eastern Mediterranean	1	0.5
Study design			
	Randomized controlled trials	7	3.3
	Longitudinal observational	94	43.9
	Retrospective cohort	91	42.5
	Surveys	12	5.6
	Case reports	6	2.8
	Qualitative	2	0.9
Sample sizes			
	0–10	16	7.5
	11–30	75	35
	31–50	49	22.9
	51–100	38	17.8
	101–200	27	12.6
	201–500	7	3.3
	501–800	2	0.9
**Participants' characteristics (*n* = 13 530)**		***n***	**%**
Age^a^ (*n* = 143 studies, 66.8%)			
Average age 6 years 6 months; range 3 years to 28 years		9346	69.1
Sex (*n* = 169 studies, 79%) (*n* = 9815 participants, 72.5%)			
	Male	6031	61.4
	Female	3784	38.6
GMFCS level (*n* = 110 studies, 51.4%) (*n* = 5454 participants, 40.3%)			
	I	342	6.3
	II	777	14.2
	III	890	16.3
	IV	319	5.9
	V	49	0.9
	Combined levels (I–III)	856	15.6
	Combined levels (I–V)	2221	40.7
Cerebral palsy subtype^b^ (*n* = 8491, 62.8%)			
(*n* = 150 studies, 72.9%)	Spastic diplegia	4382	51.6
	Spastic quadriplegia	1159	13.6
	Triplegia	216	2.5
	Hemiplegia	329	3.9
	Other (upper limb, monoplegia)	46	0.5
	Combined diplegia, quadriplegia, triplegia, hemiplegia	629	7.4
	Bilateral spastic	1097	12.9
	Unilateral spastic	10	0.1
	Spastic cerebral palsy (not specified)	2760	32.5
(*n* = 12 studies, 5.6%)	Ambulatory	619	
	Non‐ambulatory	80	
Follow‐up duration			
*n* = 120 studies	Up to 2 years	6694	49.5
*n* = 29	Up to 5 years	2048	15.1
*n* = 47	Up to 18 years	4169	30.8
*n* = 16	Into adulthood	567	4.2
*n* = 2	Not clear	52	0.4

^a^ Where the mean age and corresponding range were provided.

^b^ As reported by the original study.

Abbreviation: GMFCS, Gross Motor Function Classification System.

### Distribution of outcomes across the ICF domains

**FIGURE 1:**
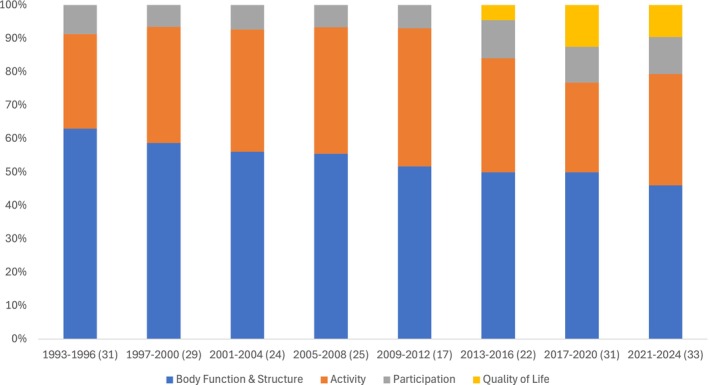
Percentage distribution of International Classification for Functioning, Disability and Health (ICF) outcomes over the years. Number of publications in each 4‐year period included in parentheses.

Ninety‐one (43%) studies reported outcomes in a single domain of the ICF, 90 (42%) included two domains, 30 (14%) reported outcomes in more than two domains, and two studies^50^, ^51^ focused on quality of life. Across all studies, the body function and structure domain was most reported (*n* = 199, 93%), followed by activity (*n* = 123, 58%) and participation (*n* = 33, 15%). Quality of life was reported in 16 (8%) studies, and four (2%) studies^50^, ^52^, ^53^, ^54^ mentioned individualized goals for SDR surgery (Appendix S3). The trends in distribution of outcomes over time showed a slight increase in participation‐based outcomes as well as the introduction of health‐related quality‐of‐life outcomes in the past decade (Figure 1). Outcomes in body function and structure are still most widely reported; however, the relative proportion is reducing. Two qualitative studies^48^, ^49^ explored parental perspectives and children's experiences on the outcomes of SDR (Appendix S3). Studies with survey designs included questions across all domains of the ICF except one survey study^41^ focusing on the caregivers' burden.

### Outcome measurement tools

**FIGURE 2:**
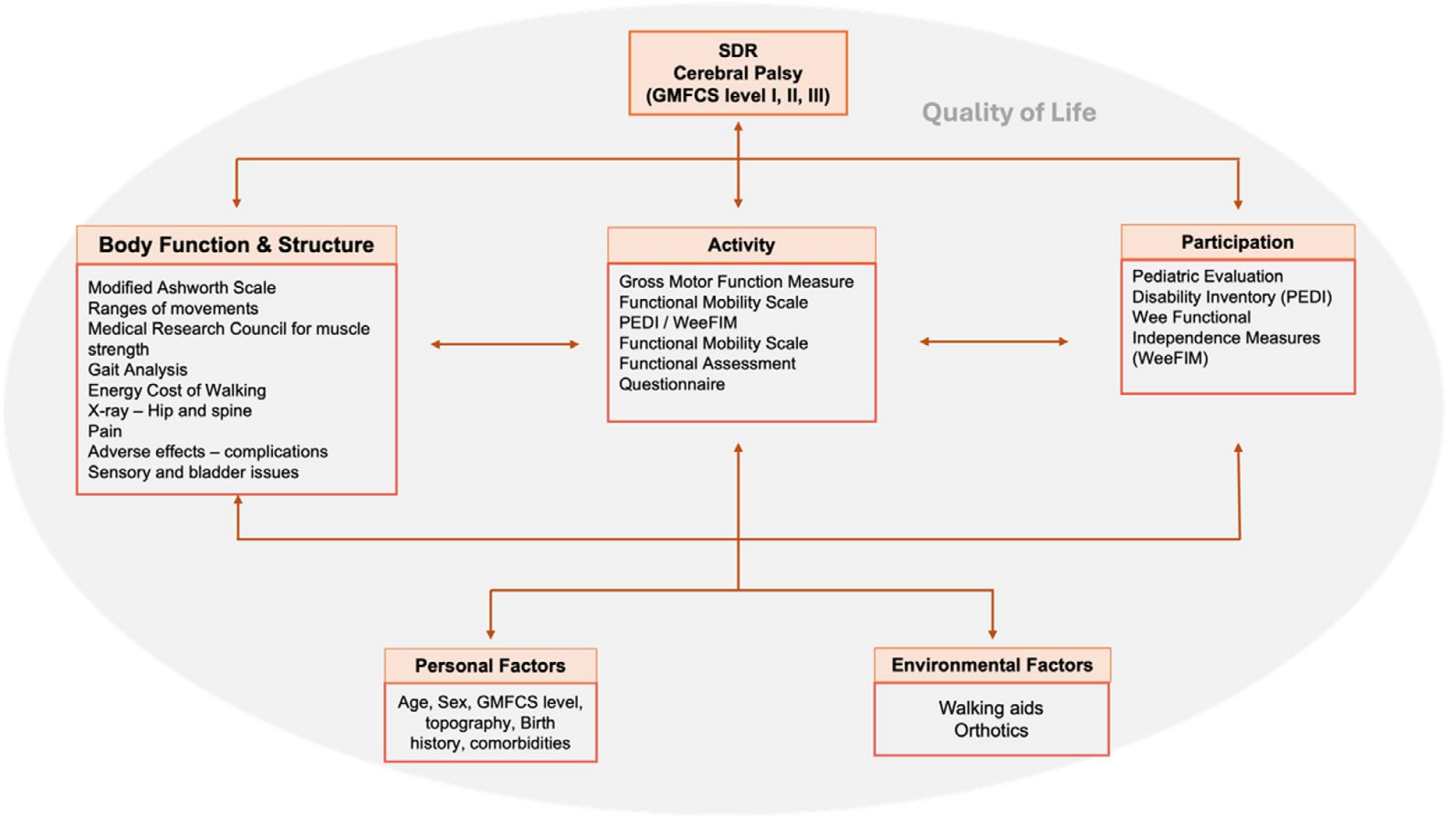
Commonly used outcome measures mapped across the International Classification for Functioning, Disability and Health (ICF). Abbreviations: GMFCS, Gross Motor Function Classification System; PEDI, Pediatric Evaluation of Disability Inventory; WeeFIM, Wee Functional Independence Measure.

A variety of outcome measurement tools (*n* = 113) were used across all ICF domains but some consistency and trends in outcomes were noted. The most frequently used outcome measures were the Modified Ashworth Scale^55^ or its variants (*n* = 91, 43%), joint range of movement (*n* = 66, 31%), instrumented gait analysis (*n* = 52, 24%), Gross Motor Function Measure^56^ (*n* = 60, 28%) in the activity domain, and Pediatric Evaluation of Disability Inventory^19^, ^57^ (*n* = 13, 6%) in the activity and participation domains (Figure 2). The distribution of identified outcomes and the measures used are mapped across the ICF domains (Appendix S4).

### Length of follow‐up

The length of follow‐up after SDR varied from immediate postoperative outcomes to long‐term follow‐up into adulthood. Most studies (*n* = 120, 56%) reported short‐term outcomes up to 2 years after SDR, with fewer undertaking longitudinal follow‐up up to 5 years (*n* = 29, 14%). Some followed patients up to 18 years (*n* = 47, 23%), or over longer terms into adulthood (*n* = 16, 8%). In two studies,^58^, ^59^ the length of follow‐up was not clear.

### Quality of life

Sixteen studies reported quality of life using validated questionnaires, including the Diener Satisfaction with Life Scale (*n* = 5),^32^, ^34^, ^35^, ^60^, ^61^ Cerebral Palsy Quality of Life questionnaire^50^, ^51^, ^62^, ^63^, ^64^ (*n* = 5), 36‐Item Short Form Health Survey^40^, ^65^, ^66^ (*n* = 3), Abbreviated WHO Quality of LIfe assessment^60^, ^61^ (*n* = 2), and opinions of patients and caregivers about aspects of their quality of life after surgery.^67^

### Goal setting and satisfaction

Four studies reported individualized goals of SDR,^50^, ^52^, ^53^, ^54^ with the Canadian Occupational Performance Measure^68^ used in three.^50^, ^53^, ^54^ Two studies described and provided examples of individualized goals in the areas of self‐care, leisure, and productivity,^50^ and personal care, functional mobility, community management, work, household management, and recreation.^53^ One study reported on the achievement of main goal areas, including improvement in comfort, orthopaedic risks, and improvements in sitting, standing, and visceral functions.^52^ Seventeen studies reported parents' or participants' satisfaction with the SDR procedure^30^, ^31^, ^32^, ^34^, ^35^, ^38^, ^39^, ^66^, ^69^, ^70^, ^71^, ^72^ using a subjective question.

### Other outcomes

Six studies reported changes in bladder function before and after SDR.^53^, ^54^, ^73^, ^74^, ^75^, ^76^ Some studies reported other outcomes often described as ‘suprasegmental’ effects of SDR. These included outcomes not directly related to the sectioning of the lumbosacral dorsal nerve root sections. Thirteen studies reported effects on upper limbs, including changes in muscle tone (*n* = 2),^77^, ^78^ movement pattern, hand function, and fine motor skills (*n* = 10) and self‐reported current manual ability using MACS^32^, ^34^, ^35^ in survey studies. The outcome measures used to assess upper limb function included the Quality of Upper Extremity Skills Test (*n* = 3)^79^ and the fine motor domain of Peabody Developmental Motor Scales (*n* = 3).^80^ Changes in eye movements,^43^, ^81^ cognitive performance,^82^ and speech^81^ were also reported.

### Adverse events

Ninety‐four studies (44%) mentioned SDR adverse events or complications, with 21 explicitly stating no complications. The most commonly reported complications were abnormal sensations in the first 6 weeks after the surgery (*n* = 34), followed by urinary complications (*n* = 35), postoperative back and leg pain (*n* = 18), and long‐term back and leg pain (*n* = 12), postoperative hypotonia or weakness (*n* = 11), constipation (*n* = 10), pulmonary complications (*n* = 10), long‐term sensory issues (*n* = 9), wound healing (*n* = 11), cerebrospinal fluid leak (*n* = 11), postoperative infections (*n* = 7), and headaches (*n* = 6). Two case‐report studies reported incidences of sudden falls^45^ and spinal cord tethering.^42^

### Additional interventions after SDR

Several studies (*n* = 54, 25%) reported on orthopaedic interventions required after SDR. The incidence of spinal deformities and hip migration after SDR was reported in 30 (14%) and 16 (6%) studies respectively.

### Contextual factors

Personal and environmental factors were not reported consistently. Most studies provided basic characteristics of preoperative patients (age, sex, baseline ambulatory status), with some (*n* = 30) providing additional demographic characteristics of the participants, such as birth history, comorbidities, and cognitive level. Studies in the adult population who had undergone SDR in childhood presented information on employment status (*n* = 8), socioeconomic status (*n* = 7), education level (*n* = 6), living situation (*n* = 5), and marital status (*n* = 4). Only eight studies reported on the use of orthotics and assistive devices.

## DISCUSSION

This scoping review identified 214 studies on SDR outcomes over the past three decades. SDR is now an established treatment for managing spasticity in children and young people with bilateral spastic CP and is available in more than 27 countries, with the number of publications reporting SDR outcomes increasing in recent years. Since the introduction of the ICF more than 20 years ago, there has been a gradual shift in CP research towards understanding the effect of interventions across all domains, including activity and participation.^10^, ^83^ However, the emphasis of SDR outcome research remains on impairment‐based outcomes in the body function and structure domain. Despite growing recognition of the importance of capturing the participation and quality‐of‐life outcomes, these are still inconsistently measured or reported in the SDR literature.

Although SDR is an invasive surgical procedure that reduces spasticity, children, young people, and families often have broader goals around function, participation, and improving family life.^50^ The relationship between spasticity and functional outcomes is complex and influenced by multiple factors, such as age at surgery, baseline gross motor ability, muscle strength, voluntary motor control, and the child's motivation and interests.^84^, ^85^ Importantly, a reduction in spasticity after SDR does not inherently result in improved function or participation. Therefore, it is essential for health‐care professionals to communicate these complexities to families, ensuring that families' expectations and goals for SDR are aligned with anticipated outcomes for each individual child, thereby supporting informed and shared decision‐making. Capturing and reporting such measures along with the other domains of the ICF^86^ is valuable, yet a significant knowledge gap persists about patient‐specific goals for SDR. Participation‐based goals and outcomes may not be achieved through spasticity reduction alone. It is likely that achieving such goals may require a targeted participation‐based approach addressing other barriers to participation.^87^ Including these outcomes can help determine how SDR and rehabilitation affect the ability of children and young people with bilateral spastic CP to engage in real‐world situations at home, school, and in the community.

We identified 113 different measures and tools used in the SDR literature across the body function and structure, activity, and participation domains. Although some quality‐of‐life measures, such as the Cerebral Palsy Quality of Life,^88^ include aspects related to the environment, none of the outcome measures used were intended to assess the influence of environmental factors specifically. The diversity in the range of outcome measures used is probably multifactorial, from the availability of standardized outcomes, feasibility (time and resources), and clinical use. Participation and contextual factors are reported less frequently, possibly because of the lack of availability of standardized outcome measures that are meaningful to families and clinicians and are sensitive enough to capture changes over time. In recent decades, there has been an increased focus on determining the effect of interventions on quality of life^89^ and identifying environmental barriers and facilitators to participation.^90^ Measures such as Participant and Environment Measure for Children and Youth^91^ and European Child Environment Questionnaire^92^ could help systematically capture the influence of environmental factors on daily activities, participation, and quality of life. Outcome measures such as the Canadian Occupational Performance Measure,^68^ Goal Attainment Scale,^93^ or Gait Outcome Assessment List^94^ can be helpful in identifying parents' and children's goals for SDR^50^ as used in other neurosurgical techniques such as deep brain stimulation and intrathecal baclofen.^95^, ^96^

Collecting outcomes longitudinally is vital for understanding changes in the functional trajectory in children and young people with bilateral spastic CP who undergo SDR in childhood. However, selecting and capturing relevant outcomes across the lifespan can be challenging owing to the lack of validated measures for adults with CP. Benner et al. highlighted the differences in the outcome measures used in children and young people and adults with CP.^97^ While impairment‐based measures (e.g. tone, range of motion, strength, gait analysis) extend into adulthood, there are no comparable standardized measures across the other ICF domains (activity, participation, environmental factors). Although the items in the outcome measures commonly used in childhood, for example the Gross Motor Function Measure, may still be relevant in capturing abilities, they are not appropriate or meaningful owing to the changing functional needs of children as they transition into their adolescent years and adulthood. Schiariti et al. developed a toolbox of standardized measures aligned with the ICF core sets for children and young people with bilateral spastic CP up to 18 years of age.^98^ However, key intervention‐specific outcomes such as pain, spasticity, or gait outcomes are not included. This mismatch between clinical and patient‐centred outcomes emphasizes the need for a core outcome set in SDR studies similar to that developed for lower‐limb orthopaedic surgery for children and young people with bilateral spastic CP.^99^ Establishing such a set would standardize assessments across centres, facilitate data pooling, and strengthen the evidence base on SDR outcomes. It could also aid decision‐making preoperatively for families and clinicians by providing more relevant information for selecting appropriate candidates for SDR intervention.

The research methodologies and study designs used in the SDR literature are predominantly quantitative observational studies, providing only one perspective, which limits a broader holistic understanding of the impact of SDR. This focus probably reflects the nature of the intervention and follow‐ups being conducted in clinical settings where the emphasis is on clinical decision‐making, which may differ from what is meaningful to children and young people with bilateral spastic CP and their families. Only two studies explored parental and children and young people perspectives on SDR outcomes.^48^, ^100^ Including these perspectives and evaluating the psychological impact can offer deeper insights into families' expectations and support decision‐making.^9^, ^49^ Clinicians should use evidence‐based measures to inform treatment decisions, monitor progress, and enable comparisons across centres and patient populations. Clear documentation of ICF personal and environmental factors can reveal patterns despite the heterogeneity in children and young people with bilateral spastic CP. Moreover, integrating participation outcomes, goal‐setting practices, or frameworks such as the F‐words can enhance communication and better align SDR intervention goals with families' priorities.^101^ Such integration, alongside clinical assessments, enables a more holistic evaluation and can help clinicians and families plan future interventions to maximize function.

Other outcomes, such as adverse effects of SDR, have been inconsistently reported across studies. These events range from immediate peri‐ or postoperative surgical complications such as infection, cerebrospinal fluid leak, bladder dysfunction, and dysesthesias to orthopaedic outcomes such as hip migration and further orthopaedic surgeries. Inconsistent language and varied descriptions across studies made synthesis challenging. Mishra et al.^102^ recently categorized SDR‐related complications as structural (e.g. hip migration, spinal deformities) and non‐structural. However, because hip dysplasia and scoliosis frequently occur in children and young people with bilateral spastic CP, irrespective of SDR, attributing these directly to SDR may be inappropriate. There is a need for further consensus and standardization in reporting adverse events.

Research on SDR outcomes has increased over time, and the reporting of research findings has evolved over the years. The findings of this scoping review should be interpreted considering the publication guidelines at the time, which may have resulted in a potential bias with favourable reporting of some outcomes. Additionally, most research comes from North America and European countries, with limited evidence from low‐ to middle‐income countries. This raises questions about the worldwide accessibility of this procedure and the choice of outcome measures. Variations in health‐care provision^103^ and treatment protocols between and within countries,^3^, ^4^ such as the timing of SDR intervention, access to and frequency of rehabilitation, and support in the community, further highlight the need to consider contextual factors when interpreting SDR outcomes.

There are some other limitations to this review. Multiple publications from the same centre may have falsely overestimated the frequency of some measures used. Although some studies referred to their previous or concurrent publications, this was not consistent across the SDR literature. Reviews, commentaries, and abstracts were excluded to reduce duplication. The reference lists of previous systematic reviews were hand‐searched, but the grey literature was not searched. This could have resulted in some omission of outcome measures reported in this scoping review. The focus was on the types of outcome and measures used rather than quality of evidence or psychometric properties of these measures. Categorizing and mapping some measures to the ICF required frequent discussions, particularly for concepts not clearly defined or coded in the ICF, such as adverse events, measures of participation, life satisfaction, and quality of life.

In conclusion, this scoping review offers a comprehensive summary of outcomes reported in the SDR literature over the past three decades. Most studies focused on outcomes in the ICF domain of body function and structure, commonly using measures such as the Modified Ashworth Scale, joint range assessments, and gait analysis across various follow‐up periods. In the activity domain, the Gross Motor Function Measure remains the most frequently used outcome measure. A smaller number of studies, particularly those with longer‐term follow‐up into adulthood or survey‐based designs, have reported contextual factors and outcomes related to participation and quality of life. The review highlights several gaps that have implications for both clinical practice and future research. Greater consistency in language and systematic reporting of contextual factors and adverse events will improve cross‐study comparisons and enhance generalizability. To capture meaningful outcomes across the lifespan, future research should use broader study designs, such as qualitative, mixed‐methods, or participatory approaches, incorporating perspectives from children, young people, and families. The development of a core outcome set, informed by all stakeholders, would support greater consistency in SDR research and clinical practice.

## CONFLICT OF INTEREST STATEMENT

The authors have stated that they had no interests that might be perceived as posing a conflict or bias.

## Supporting information


**Appendix S1:** Search terms and search strategy for all six databases.

**Appendix S2:** Data extraction instrument.

**Appendix S3:** Visual representation of data.

**Appendix S4:** Outcome domains and outcome measures used in the literature and ICF coding.

**Table S1:** Excluded articles.

**Table S2:** Included studies.

**Figure S1:** PRISMA flow diagram.

## Data Availability

The data that supports the findings of this study are available in the supplementary material of this article.

## References

[1] Park TS , Johnston JM . Surgical techniques of selective dorsal rhizotomy for spastic cerebral palsy. Technical note. Neurosurgical focus. 2006;21(2):e7. 16918228

[2] NICE . Selective dorsal rhizotomy for spasticity in cerebral palsy. National Institute for Health and Care Excellence; 2010. p. Interventional procedures guidance.

[3] Nicolini‐Panisson RD , Tedesco AP , Folle MR , Donadio MVF . Selective Dorsal Rhizotomy in Cerebral Palsy: Selection Criteria and Postoperative Physical Therapy Protocols. Rev Paul Pediatr. 2018;36(1):9. 10.1590/1984-0462/;2018;36;1;00005 PMC5849370 29412426

[4] Grunt S , Fieggen AG , Vermeulen RJ , Becher JG , Langerak NG . Selection criteria for selective dorsal rhizotomy in children with spastic cerebral palsy: a systematic review of the literature. Developmental Medicine and Child Neurology. 2014;56(4):302–12. 24106928 10.1111/dmcn.12277

[5] Grunt S , Becher JG , Vermeulen RJ . Long‐term outcome and adverse effects of selective dorsal rhizotomy in children with cerebral palsy: a systematic review. Developmental Medicine and Child Neurology. 2011;53(6):490–8. 21518341 10.1111/j.1469-8749.2011.03912.x

[6] Tedroff K , Hagglund G , Miller F . Long‐term effects of selective dorsal rhizotomy in children with cerebral palsy: a systematic review. Developmental Medicine and Child Neurology. 2020;62(5):554–62. 31342516 10.1111/dmcn.14320 PMC7187377

[7] Bolster EAM , Van Schie PEM , Becher JG , Van Ouwerkerk WJR , Strijers RLM , Vermeulen RJ . Long‐term effect of selective dorsal rhizotomy on gross motor function in ambulant children with spastic bilateral cerebral palsy, compared with reference centiles. Developmental Medicine and Child Neurology. 2013;55(7):610–6. 23557106 10.1111/dmcn.12148

[8] Iorio‐Morin C , Yap R , Dudley RWR , Poulin C , Cantin M‐A , Benaroch TE , et al. Selective Dorsal Root Rhizotomy for Spastic Cerebral Palsy: A Longitudinal Case‐Control Analysis of Functional Outcome. Neurosurgery. 2020;87(2):186–92. 31620799 10.1093/neuros/nyz422

[9] Waite G , Chugh D , Cawker S , Oulton K , Wray J , Harniess P . ‘Wanting no regrets’: Parental decision making around selective dorsal rhizotomy. Child Care Health and Development. 2023;49(2):382–91. 36057954 10.1111/cch.13056

[10] World Health Organization . Towards a Common Language for Functioning, Disability and Health: ICF The International Classification of Functioning, Disability and Health. 2002.

[11] Rosenbaum P , Stewart D . The World Health Organization International Classification of Functioning, Disability, and Health: a model to guide clinical thinking, practice and research in the field of cerebral palsy. Semin Pediatr Neurol. 2004;11(1):5–10. 15132248 10.1016/j.spen.2004.01.002

[12] King S , Teplicky R , King G , Rosenbaum P . Family‐centered service for children with cerebral palsy and their families: a review of the literature. Semin Pediatr Neurol. 2004;11(1):78–86. 15132256 10.1016/j.spen.2004.01.009

[13] McDougall J , Wright V , DeWit D , Miller L . ICF‐based functional components and contextual factors as correlates of perceived quality of life for youth with chronic conditions. Disabil Rehabil. 2014;36(25):2143–51. 24575718 10.3109/09638288.2014.892642 PMC4364266

[14] Reinhardt JD , Miller J , Stucki G , Sykes C , Gray DB . Measuring impact of environmental factors on human functioning and disability: a review of various scientific approaches. Disabil Rehabil. 2011;33(23–24):2151–65. 21548824 10.3109/09638288.2011.573053

[15] Peters MDJ , Marnie C , Tricco AC , Pollock D , Munn Z , Alexander L , et al. Updated methodological guidance for the conduct of scoping reviews. JBI Evid Synth. 2020;18(10):2119–26. 33038124 10.11124/JBIES-20-00167

[16] Chugh D ME , Waite G , Gimeno H . Outcome measures used for ambulant children with spastic cerebral palsy who have undergone selective dorsal rhizotomy: a scoping review protocol. Open Science Framework2024 [

[17] McGowan J , Sampson M , Salzwedel DM , Cogo E , Foerster V , Lefebvre C . PRESS Peer Review of Electronic Search Strategies: 2015 Guideline Statement. J Clin Epidemiol. 2016;75:40–6. 27005575 10.1016/j.jclinepi.2016.01.021

[18] Oxford Centre for Evidence‐based Medicine Scale [Available from: https://www.cebm.ox.ac.uk/resources/levels‐of‐evidence/oxford‐centre‐for‐evidence‐based‐medicine‐levels‐of‐evidence‐march‐2009.

[19] Haley S , Coster, W ., Ludlow, L ., Haltiwanger, J ., & Andrellos, P . Pediatric evaluation of disability (PEDI). Boston: New England Center Hospitals/PEDI Research Group. 1992.

[20] Ottenbacher KJ , Msall ME , Lyon NR , Duffy LC , Granger CV , Braun S . Interrater agreement and stability of the Functional Independence Measure for Children (WeeFIM): use in children with developmental disabilities. Arch Phys Med Rehabil. 1997;78(12):1309–15. 9421983 10.1016/s0003-9993(97)90302-6

[21] Ouzzani M , Hammady H , Fedorowicz Z , Elmagarmid A . Rayyan‐a web and mobile app for systematic reviews. Syst Rev. 2016;5(1):210. 27919275 10.1186/s13643-016-0384-4 PMC5139140

[22] Tricco AC , Lillie E , Zarin W , O'Brien KK , Colquhoun H , Levac D , et al. PRISMA Extension for Scoping Reviews (PRISMA‐ScR): Checklist and Explanation. Ann Intern Med. 2018;169(7):467–73. 30178033 10.7326/M18-0850

[23] Steinbok P , Reiner AM , Beauchamp R , Armstrong RW , Cochrane DD , Kestle J . A randomized clinical trial to compare selective posterior rhizotomy plus physiotherapy with physiotherapy alone in children with spastic diplegic cerebral palsy. Developmental medicine and child neurology. 1997;39(3):178–84. 9112967 10.1111/j.1469-8749.1997.tb07407.x

[24] Steinbok P , Reiner A , Kestle JR . Therapeutic electrical stimulation following selective posterior rhizotomy in children with spastic diplegic cerebral palsy: a randomized clinical trial. Developmental medicine and child neurology. 1997;39(8):515–20. 9295846 10.1111/j.1469-8749.1997.tb07479.x

[25] Wright FV , Sheil EM , Drake JM , Wedge JH , Naumann S . Evaluation of selective dorsal rhizotomy for the reduction of spasticity in cerebral palsy: a randomized controlled tria. Developmental medicine and child neurology. 1998;40(4):239–47. 9593495 10.1111/j.1469-8749.1998.tb15456.x

[26] McLaughlin JF , Bjornson KF , Astley SJ , Graubert C , Hays RM , Roberts TS , et al. Selective dorsal rhizotomy: efficacy and safety in an investigator‐masked randomized clinical trial. Developmental Medicine & Child Neurology. 1998;40(4):220–32. 9593493 10.1111/j.1469-8749.1998.tb15454.x

[27] Graubert C , Song KM , McLaughlin JF , Bjornson KF . Changes in gait at 1 year post‐selective dorsal rhizotomy: Results of a prospective randomized study. Journal of Pediatric Orthopaedics. 2000;20(4):496–500. 10912607

[28] Abd‐Elmonem AM , Ali HA , Saad‐Eldien SS , Rabiee A , Abd El‐Nabie WA . Effect of physical training on motor function of ambulant children with diplegia after selective dorsal rhizotomy: A randomized controlled study. Neurorehabilitation. 2023;53(4):547–56. 38143389 10.3233/NRE-230098

[29] Mu XH , Xu L , Xu SG , Cao X , Zhang P , Zheng CY , et al. Application of exercise therapy on rehabilitation after selective posterior rhizotomy (SPR) in children with cerebral palsy. Zhongguo gu shang = China journal of orthopaedics and traumatology. 2009;22(9):674 EP ‐ 6. 19817198

[30] O'Brien DF , Park TS , Puglisi JA , Collins DR , Leuthardt EC . Effect of selective dorsal rhizotomy on need for orthopedic surgery for spastic quadriplegic cerebral palsy: long‐term outcome analysis in relation to age. Journal of Neurosurgery. 2004;101(1):59–63. 16206973 10.3171/ped.2004.101.2.0059

[31] Langerak NG , Hillier SL , Verkoeijen PP , Peter JC , Fieggen AG , Vaughan CL . LEVEL OF ACTIVITY AND PARTICIPATION IN ADULTS WITH SPASTIC DIPLEGIA 17–26 YEARS AFTER SELECTIVE DORSAL RHIZOTOMY. Journal of Rehabilitation Medicine. 2011;43(4):330–7. 21327322 10.2340/16501977-0669

[32] Hurvitz EA , Marciniak CM , Daunter AK , Haapala HJ , Stibb SM , McCormick SF , et al. Functional outcomes of childhood dorsal rhizotomy in adults and adolescents with cerebral palsy Clinical article. Journal of Neurosurgery‐Pediatrics. 2013;11(4):380–8. 23394352 10.3171/2013.1.PEDS12311

[33] Daunter AK , Kratz AL , Hurvitz EA . Long‐term impact of childhood selective dorsal rhizotomy on pain, fatigue, and function: a case–control study. Developmental Medicine and Child Neurology. 2017;59(10):1089–95. 28617943 10.1111/dmcn.13481 PMC5610610

[34] Park TS , Edwards C , Liu EL , Walter DM , Dobbs MB . Beneficial Effects of Childhood Selective Dorsal Rhizotomy in Adulthood. Cureus Journal of Medical Science. 2017;9(3). 10.7759/cureus.1077 PMC5382010 28401027

[35] Park TS , Liu JL , Edwards C , Walter DM , Dobbs MB . Functional Outcomes of Childhood Selective Dorsal Rhizotomy 20 to 28 Years Later. Cureus Journal of Medical Science. 2017;9(5). 10.7759/cureus.1256 PMC5473717 28649479

[36] Park TS , Miller BA , Cho J . Simultaneous Selective Dorsal Rhizotomy and Baclofen Pump Removal Improve Ambulation in Patients with Spastic Cerebral Palsy. Cureus Journal of Medical Science. 2018;10(6). 10.7759/cureus.2791 PMC6089480 30112267

[37] Veerbeek BE , Lamberts RP , Fieggen AG , Verkoeijen PPJL , Langerak NG . Daily activities, participation, satisfaction, and functional mobility of adults with cerebral palsy more than 25 years after selective dorsal rhizotomy: a long‐term follow‐up during adulthood. Disability and Rehabilitation. 2021;43(15):2191–9. 31815556 10.1080/09638288.2019.1695001

[38] Park TS , Joh S , Walter DM , Dobbs MB . Selective Dorsal Rhizotomy for the Treatment of Spastic Triplegic Cerebral Palsy. Cureus Journal of Medical Science. 2020;12(7). 10.7759/cureus.9204 PMC7430432 32821558

[39] Park TS , Joh S , Walter DM , Meyer NL . Parent‐Reported Outcomes of Early Childhood Selective Dorsal Rhizotomy for the Treatment of Spastic Diplegia. Cureus Journal of Medical Science. 2021;13(6). 10.7759/cureus.15530 PMC8265858 34268050

[40] Veerbeek BE , Lamberts RP , Kosel E , Fieggen AG , Langerak NG . More than 25 years after selective dorsal rhizotomy: physical status, quality of life, and levels of anxiety and depression in adults with cerebral palsy. Journal of Neurosurgery. 2022;136(3):689–98. 34507281 10.3171/2021.3.JNS204096

[41] de Monaco BA , Candido AAD , Teixeira MJ , Alho EJL . Impact of selective dorsal rhizotomy to cerebral palsy children caregivers' burden. Childs Nervous System. 2024;40(5):1461–9. 10.1007/s00381-024-06291-1 38252157

[42] Gooch JL , Walker ML . Spinal stenosis after total lumbar laminectomy for selective dorsal rhizotomy. Pediatric Neurosurgery. 1996;25(1):28–30. 9055331 10.1159/000121092

[43] Horínek D , Hoza D , Cerny R , Vyhnálek M , Sturm D , Bojar M , et al. Two cases of improvement of smooth pursuit eye movements after selective posterior rhizotomy. Childs Nervous System. 2008;24(11):1283–8. 10.1007/s00381-008-0673-x 18688617

[44] Spijker M , Strijers RLM , van Ouwerkerk WJR , Becher JG . Disappearance of Spasticity After Selective Dorsal Rhizotomy Does Not Prevent Muscle Shortening in Children With Cerebral Palsy: A Case Report. Journal of Child Neurology. 2009;24(5):625–7. 19151363 10.1177/0883073808325652

[45] Grootveld LR , van Schie PE , Buizer AI , Jeroen Vermeulen R , van Ouwerkerk WJ , Strijers RL , et al. Sudden falls as a persistent complication of selective dorsal rhizotomy surgery in children with bilateral spasticity: report of 3 cases. J Neurosurg Pediatr. 2016;18(2):192–5. 27104630 10.3171/2016.2.PEDS15527

[46] Gimarc K , Yandow S , Browd S , Leibow C , Pham K . Combined Selective Dorsal Rhizotomy and Single‐Event Multilevel Surgery in a Child with Spastic Diplegic Cerebral Palsy: A Case Report. Pediatric Neurosurgery. 2021. 10.1159/000517756 34384084

[47] Belanger K , McKay W , Oleszek J , Graber S , Wilkinson C . Spinal cord tethering after selective dorsal rhizotomy below the conus medullaris. Child's Nervous System. 2022;38(11):2129–32. 10.1007/s00381-022-05633-1 35978197

[48] Eliasson AC , Öhrvall AM , Borell L . Parents' perspectives of changes in movement affecting daily life following selective dorsal rhizotomy in children with cerebral palsy. Physical and Occupational Therapy in Pediatrics. 2000;19(3–4):91–109.

[49] Chugh D , Waite G , Harniess P , Oulton K , Wray J , Cawker S . ‘I Didn't Know What Was Going to Happen’: Children's and Young People's Experiences and Their Involvement Before and After Selective Dorsal Rhizotomy. Physical & Occupational Therapy in Pediatrics. 2024. 10.1080/01942638.2024.2323192 39118452

[50] Lewis JA , Bear N , Smith N , Baker F , Lee OS , Wynter M , et al. Goal setting, goal attainment and quality of life of children following selective dorsal rhizotomy. Child: Care, Health and Development. 2023;49(4):760–8. 36513964 10.1111/cch.13090

[51] Robins JMW , Boyle A , McCune K , Lodh R , Goodden JR . Quality of life after selective dorsal rhizotomy: an assessment of family‐reported outcomes using the CPQoL questionnaire. Childs Nervous System. 2020;36(9):1977–83. 10.1007/s00381-020-04546-1 32095868

[52] Hodgkinson I , Berard C , Jindrich ML , Sindou M , Mertens P , Berard J . Selective dorsal rhizotomy in children with cerebral palsy. Results in 18 cases at one year postoperatively. Stereotactic and functional neurosurgery. 1997;69(1–4 Pt 2):259–67. 9711764 10.1159/000099885

[53] Chan SHS , Yam KY , Yiu‐Lau BPH , Poon CYC , Chan NNC , Cheung HM , et al. Selective dorsal rhizotomy in Hong Kong: Multidimensional outcome measures. Pediatric Neurology. 2008;39(1):22–32. 18555169 10.1016/j.pediatrneurol.2008.03.017

[54] He ZX , Wong ST , Law HY , Lao LMM , Chan KFH , Chan NCN , et al. Multidimensional Outcomes of Selective Dorsal Rhizotomy for Children With Spastic Cerebral Palsy: Single‐Level Laminectomy vs Multiple‐Level Laminotomy Techniques. Neurosurgery. 2022;91(3):513–24. 35881026 10.1227/neu.0000000000002036

[55] Bohannon RW , Smith MB . Interrater reliability of a modified Ashworth scale of muscle spasticity. Phys Ther. 1987;67(2):206–7. 3809245 10.1093/ptj/67.2.206

[56] Russell DJ , Avery LM , Rosenbaum PL , Raina PS , Walter SD , Palisano RJ . Improved scaling of the gross motor function measure for children with cerebral palsy: Evidence of reliability and validity. Physical Therapy. 2000;80(9):873–85. 10960935

[57] Haley SM , Coster WJ , Dumas HM , Fragala‐Pinkham MA , Kramer J , Ni P , et al. Accuracy and precision of the Pediatric Evaluation of Disability Inventory computer‐adaptive tests (PEDI‐CAT). Dev Med Child Neurol. 2011;53(12):1100–6. 22077695 10.1111/j.1469-8749.2011.04107.x PMC3638866

[58] Shuman BR , Goudriaan M , Desloovere K , Schwartz MH , Steele KM . Muscle synergies demonstrate only minimal changes after treatment in cerebral palsy. Journal of Neuroengineering and Rehabilitation. 2019;16. 10.1186/s12984-019-0502-3 PMC6441188 30925882

[59] Tichy M , Kraus J , Horinek D , Vaculik M . Selective posterior rhizotomy in the treatment of cerebral palsy, first experience in Czech Republic. Bratislavské lekárske listy. 2003;104(2):54–8. 12839212

[60] Munger ME , Aldahondo N , Krach LE , Novacheck TF , Schwartz MH . Long‐term outcomes after selective dorsal rhizotomy: a retrospective matched cohort study. Developmental Medicine and Child Neurology. 2017;59(11):1196–203. 28786493 10.1111/dmcn.13500

[61] MacWilliams BA , McMulkin ML , Duffy EA , Munger ME , Chen BPJ , Novacheck TF , et al. Long‐term effects of spasticity treatment, including selective dorsal rhizotomy, for individuals with cerebral palsy. Developmental Medicine and Child Neurology. 2022;64(5):561–8. 34755903 10.1111/dmcn.15075

[62] Summers J , Coker B , Eddy S , Elstad M , Bunce C , Bourmpaki E , et al. Selective dorsal rhizotomy in ambulant children with cerebral palsy: an observational cohort study. Lancet Child & Adolescent Health. 2019;3(7):455–62. 31047843 10.1016/S2352-4642(19)30119-1 PMC7153769

[63] Gillespie CS , George AM , Hall B , Toh S , Islim AI , Hennigan D , et al. The effect of GMFCS level, age, sex, and dystonia on multi‐dimensional outcomes after selective dorsal rhizotomy: prospective observational study. Childs Nervous System. 2021;37(5):1729–40. 10.1007/s00381-021-05076-0 PMC8084767 33599808

[64] Chow CP , Wong LY , Poon CYC , Yiu BPH , Wong TPS , Wong M , et al. Functional outcome after selective dorsal rhizotomy: a retrospective case control study. Childs Nervous System. 2024;40(3):625–34. 10.1007/s00381-023-06213-7 37979014

[65] Tedroff K , Löwing K , Åström E . A prospective cohort study investigating gross motor function, pain, and health‐related quality of life 17 years after selective dorsal rhizotomy in cerebral palsy. Developmental Medicine and Child Neurology. 2015;57(5):484–90. 25523506 10.1111/dmcn.12665

[66] Park TS , Joh S , Walter DM , Meyer NL , Dobbs MB . Selective Dorsal Rhizotomy for the Treatment of Spastic Hemiplegic Cerebral Palsy. Cureus Journal of Medical Science. 2020;12(8). 10.7759/cureus.9605 PMC7479993 32923208

[67] Santos MV , Carneiro VM , Oliveira PNBGC , Caldas CAT , MacHado HR . Surgical results of selective dorsal rhizotomy for the treatment of spastic cerebral palsy. Journal of Pediatric Neurosciences. 2021;16(1):24–9. 34316304 10.4103/jpn.JPN_26_20 PMC8276966

[68] Law M , Baptiste S , McColl M , Opzoomer A , Polatajko H , Pollock N . The Canadian occupational performance measure: an outcome measure for occupational therapy. Can J Occup Ther. 1990;57(2):82–7. 10104738 10.1177/000841749005700207

[69] O'Brien DF , Tae SP , Puglisi JA , Collins DR , Leuthardt EC , Leonard JR . Orthopedic surgery after selective dorsal rhizotomy for spastic diplegia in relation to ambulatory status and age. Journal of Neurosurgery. 2005;103 PEDIATRICS(SUPPL. 1):5–9. 16121998 10.3171/ped.2005.103.1.0005

[70] Langerak NG , Lamberts RP , Fieggen AG , Peter JC , van der Merwe L , Peacock WJ , et al. A prospective gait analysis study in patients with diplegic cerebral palsy 20 years after selective dorsal rhizotomy. Journal of Neurosurgery‐Pediatrics. 2008;1(3):180–6. 18352761 10.3171/PED/2008/1/3/180

[71] Kan P , Gooch J , Amini A , Ploeger D , Grams B , Oberg W , et al. Surgical treatment of spasticity in children: comparison of selective dorsal rhizotomy and intrathecal baclofen pump implantation. Childs Nervous System. 2008;24(2):239–43. 10.1007/s00381-007-0457-8 17805547

[72] van Schie PEM , Schothorst M , Dallmeijer AJ , Vermeulen RJ , van Ouwerkerk WJR , Strijers RLM , et al. Short‐ and long‐term effects of selective dorsal rhizotomy on gross motor function in ambulatory children with spastic diplegia Clinical article. Journal of Neurosurgery‐Pediatrics. 2011;7(5):557–62. 21529199 10.3171/2011.2.PEDS10452

[73] Sweetser PM , Badell A , Schneider S , Badlani GH . Effects of sacral dorsal rhizotomy on bladder function in patients with spastic cerebral palsy. Neurourology and Urodynamics. 1995;14(1):57–64. 7742850 10.1002/nau.1930140110

[74] Houle AM , Vernet O , Jednak R , Salle JLP , Farmer JP . Bladder function before and after selective dorsal rhizotomy in children with cerebral palsy. Journal of Urology. 1998;160(3):1088–91. 9719282 10.1097/00005392-199809020-00032

[75] Kim DS , Choi JU , Yang KH , Park CI , Park ES . Selective posterior rhizotomy for lower extremity spasticity: How much and which of the posterior rootlets should be cut? Surgical Neurology. 2002;57(2):87–93. 11904198 10.1016/s0090-3019(01)00680-2

[76] Chiu PKF , Yam KY , Lam TY , Cheng CH , Yu C , Li ML , et al. Does selective dorsal rhizotomy improve bladder function in children with cerebral palsy? International Urology and Nephrology. 2014;46(10):1929–33. 24973204 10.1007/s11255-014-0770-6

[77] Lewin JE , Mix CM , Gaebler‐Spira D . Self‐help and upper extremity changes in 36 children with cerebral palsy subsequent to selective posterior rhizotomy and intensive occupational and physical therapy. Physical and Occupational Therapy in Pediatrics. 1994;13(3):25–42.

[78] Buckon CE , Sienko Thomas S , Aiona MD , Piatt JH . Assessment of upper‐extremity function in children with spastic diplegia before and after selective dorsal rhizotomy. Developmental medicine and child neurology. 1996;38(11):967–75. 8913178 10.1111/j.1469-8749.1996.tb15057.x

[79] DeMatteo C , Law M , Russell D , Pollock N , Rosenbaum P , & Walter S . The Reliability and Validity of the Quality of Upper Extremity Skills Test. Physical & Occupational Therapy In Pediatrics. 1993;18(2):1–18.

[80] Folio M , Fewell R . Peabody Developmental Motor Scales and Activity Cards. Austin: PRO‐ED; 1983.

[81] Hoza D , Lastovka M , Bojar M , Cerny R , Sturm D , Kraus J , et al. An Improvement in Smooth Pursuit Eye Movements and Phonation Following Selective Dorsal Rhizotomy. Ceska a Slovenska Neurologie a Neurochirurgie. 2009;72(4):378–82.

[82] Craft S , Park TS , White DA , Schatz J , Noetzel M , Arnold S . Changes in cognitive performance in children with spastic diplegic cerebral palsy following selective dorsal rhizotomy. Pediatric Neurosurgery. 1995;23(2):68–75. 8555098 10.1159/000120939

[83] Jackman M , Sakzewski L , Morgan C , Boyd RN , Brennan SE , Langdon K , et al. Interventions to improve physical function for children and young people with cerebral palsy: international clinical practice guideline. Dev Med Child Neurol. 2022;64(5):536–49. 34549424 10.1111/dmcn.15055

[84] Wright FV , Rosenbaum PL , Goldsmith CH , Law M , Fehlings DL . How do changes in body functions and structures, activity, and participation relate in children with cerebral palsy? Dev Med Child Neurol. 2008;50(4):283–9. 18312423 10.1111/j.1469-8749.2008.02037.x

[85] Funk JF , Panthen A , Bakir MS , Gruschke F , Sarpong A , Wagner C , et al. Predictors for the benefit of selective dorsal rhizotomy. Research in Developmental Disabilities. 2015;37:127–34. 25460226 10.1016/j.ridd.2014.11.012

[86] Imms C , Granlund M , Wilson PH , Steenbergen B , Rosenbaum PL , Gordon AM . Participation, both a means and an end: a conceptual analysis of processes and outcomes in childhood disability. Dev Med Child Neurol. 2017;59(1):16–25. 27640996 10.1111/dmcn.13237

[87] Anaby D , Avery L , Gorter JW , Levin MF , Teplicky R , Turner L , et al. Improving body functions through participation in community activities among young people with physical disabilities. Dev Med Child Neurol. 2020;62(5):640–6. 31670397 10.1111/dmcn.14382

[88] Waters E , Davis E , Mackinnon A , Boyd R , Graham HK , Kai Lo S , et al. Psychometric properties of the quality of life questionnaire for children with CP. Dev Med Child Neurol. 2007;49(1):49–55. 17209977 10.1017/s0012162207000126.x

[89] McDougall J , Wright V , Schmidt J , Miller L , Lowry K . Applying the ICF framework to study changes in quality‐of‐life for youth with chronic conditions. Dev Neurorehabil. 2011;14(1):41–53. 21034288 10.3109/17518423.2010.521795 PMC4245180

[90] Law M , Petrenchik T , King G , Hurley P . Perceived environmental barriers to recreational, community, and school participation for children and youth with physical disabilities. Arch Phys Med Rehabil. 2007;88(12):1636–42. 18047879 10.1016/j.apmr.2007.07.035

[91] Coster W , Bedell G , Law M , Khetani MA , Teplicky R , Liljenquist K , et al. Psychometric evaluation of the Participation and Environment Measure for Children and Youth. Dev Med Child Neurol. 2011;53(11):1030–7. 22014322 10.1111/j.1469-8749.2011.04094.x

[92] Dickinson HO , Colver A , Sparcle G . Quantifying the physical, social and attitudinal environment of children with cerebral palsy. Disabil Rehabil. 2011;33(1):36–50. 20455710 10.3109/09638288.2010.485668

[93] Turner‐Stokes L. Goal attainment scaling (GAS) in rehabilitation: a practical guide. Clin Rehabil. 2009;23(4):362–70. 19179355 10.1177/0269215508101742

[94] Thomason P , Tan A , Donnan A , Rodda J , Graham HK , Narayanan U . The Gait Outcomes Assessment List (GOAL): validation of a new assessment of gait function for children with cerebral palsy. Dev Med Child Neurol. 2018;60(6):618–23. 29573409 10.1111/dmcn.13722

[95] Gimeno H , Tustin K , Lumsden D , Ashkan K , Selway R , Lin JP . Evaluation of functional goal outcomes using the Canadian Occupational Performance Measure (COPM) following Deep Brain Stimulation (DBS) in childhood dystonia. Eur J Paediatr Neurol. 2014;18(3):308–16. 24461258 10.1016/j.ejpn.2013.12.010

[96] Bonouvrie LA , Haberfehlner H , Becher JG , Vles JSH , Vermeulen RJ , Buizer AI , et al. Attainment of personal goals in the first year of intrathecal baclofen treatment in dyskinetic cerebral palsy: a prospective cohort study. Disabil Rehabil. 2023;45(8):1315–22. 35387541 10.1080/09638288.2022.2057600

[97] Benner JL , Noten S , Limsakul C , Van Der Slot WMA , Stam HJ , Selb M , et al. Outcomes in adults with cerebral palsy: systematic review using the International Classification of Functioning, Disability and Health. Dev Med Child Neurol. 2019;61(10):1153–61. 30985004 10.1111/dmcn.14247

[98] Schiariti V , Tatla S , Sauve K , O'Donnell M . Toolbox of multiple‐item measures aligning with the ICF Core Sets for children and youth with cerebral palsy. Eur J Paediatr Neurol. 2017;21(2):252–63. 27864012 10.1016/j.ejpn.2016.10.007

[99] Almoajil H , Hopewell S , Dawes H , Toye F , Theologis T . A core outcome set for lower limb orthopaedic surgery for children with cerebral palsy: An international multi‐stakeholder consensus study. Dev Med Child Neurol. 2023;65(2):254–63. 35869637 10.1111/dmcn.15351 PMC10084115

[100] Chugh D , Waite G , Cawker S , Harniess P , Wray J , Oulton K . Children and young people's perspectives on decision‐making and their experiences around selective dorsal rhizotomy. Developmental Medicine and Child Neurology. 2021;63(SUPPL 2):33.

[101] Rosenbaum P , Gorter JW . The ‘F‐words’ in childhood disability: I swear this is how we should think! Child Care Health Dev. 2012;38(4):457–63. 22040377 10.1111/j.1365-2214.2011.01338.x

[102] Mishra D , Barik S , Raj V , Kandwal P . A systematic review of complications following selective dorsal rhizotomy in cerebral palsy. Neurochirurgie. 2023;69(3). 10.1016/j.neuchi.2023.101425 36828056

[103] Mwangi LW , Abuga JA , Cottrell E , Kariuki SM , Kinyanjui SM , Newton CR . Barriers to access and utilization of healthcare by children with neurological impairments and disability in low‐and middle‐income countries: a systematic review. Wellcome Open Res. 2021;6:61. 35299711 10.12688/wellcomeopenres.16593.1 PMC8902259

