# Supplementary material for: Outcomes of selective dorsal rhizotomy in ambulatory children and young people with cerebral palsy: A scoping review
Source: Dev Med Child Neurol. 2025 Sep 19;68(2):175–86. doi: 10.1111/dmcn.16496 (PMC12766555; doi:10.1111/dmcn.16496)
Supplement: Supplementary file 1 — Appendix S1: Search terms and search strategy for all six databases. [file DMCN-68-175-s005.docx]

**Appendix S1: Search terms and Search Strategy for all six databases**

| **Search Terms** | **PRESS checklist- McGowan Jo Clinical epidemiology 2016** |
| --- | --- |
| **Population** | Cerebral Pals*, Spastic*, CP, |
|  | child* (subject heading- Child, infant*, adolescen*), Teen*, Young people |
| Concept | Rhizotom* (Selective Dorsal Rhizotomy/Selective Posterior Rhizotomy  /Functional Posterior Rhizotomy)  SDR/SPR/FPR |

Search terms were retrieved and rerun in March 2025 as per the reviewers’ feedback.

PRISMA Scoping review chart included results from the previous search (July 2024)

**Cochrane library**


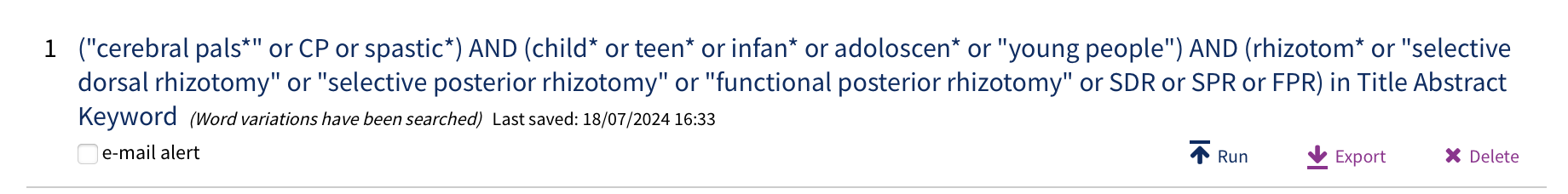


**Scopus**
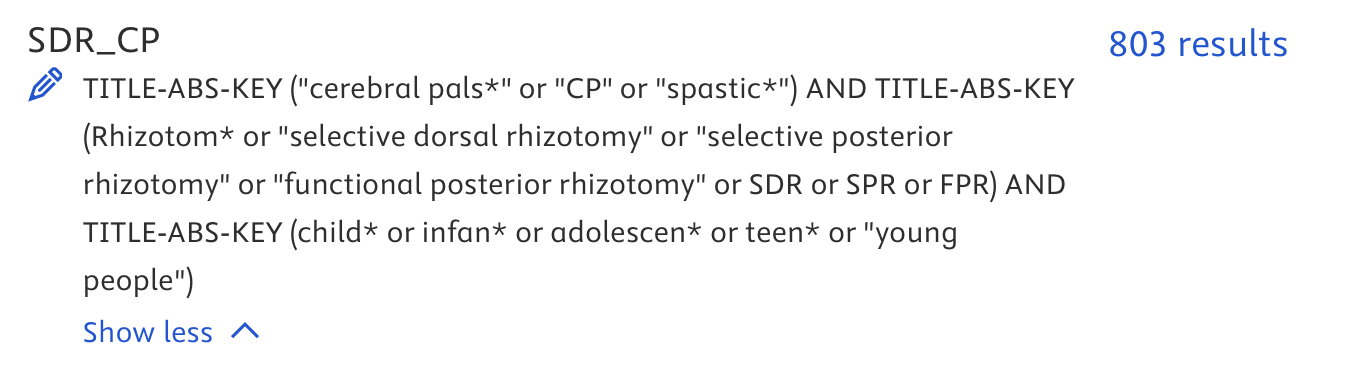


**Web of Science**


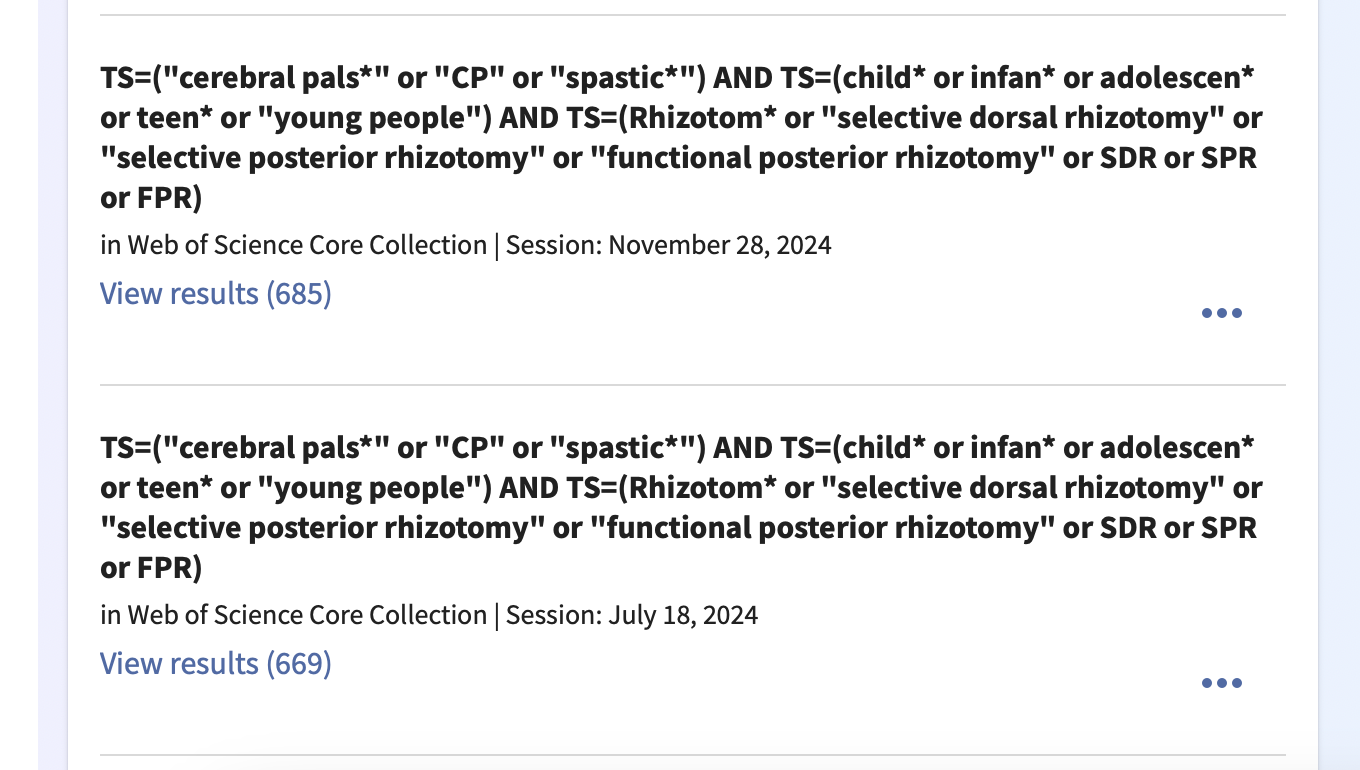


**EMBASE**

Embase


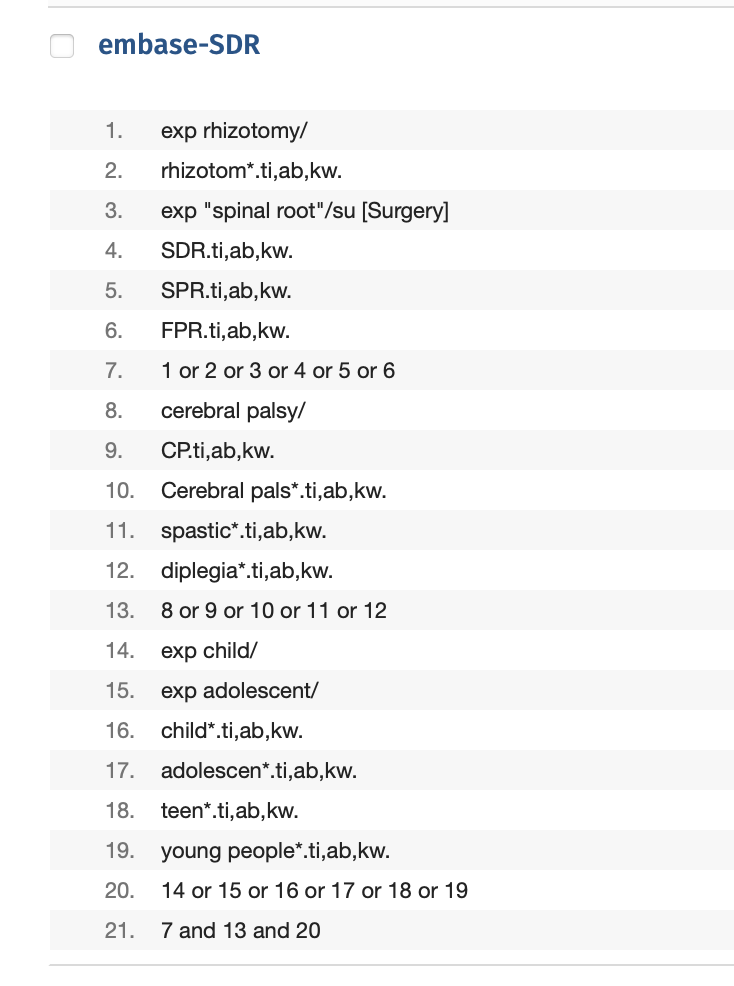


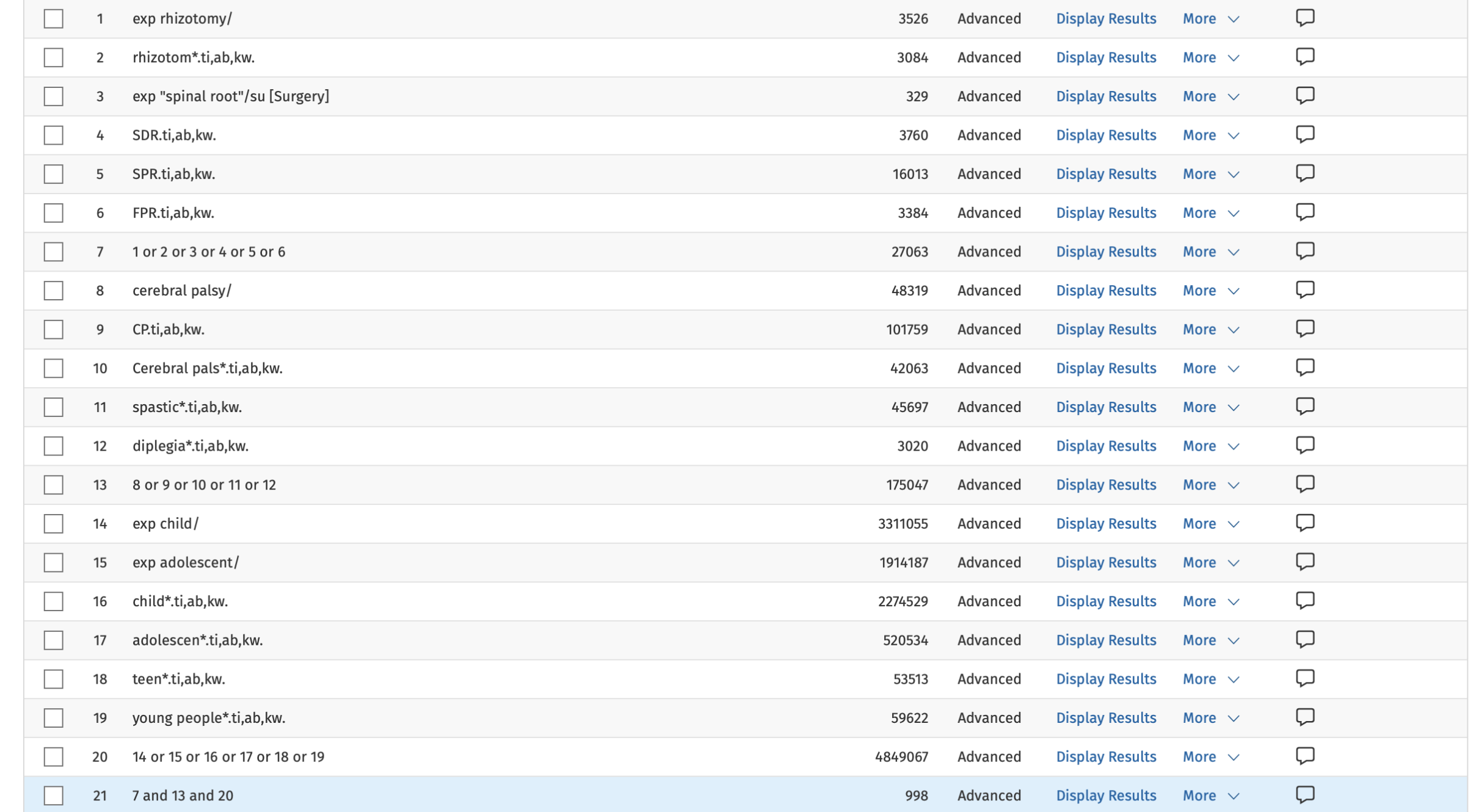


**MEDLINE**


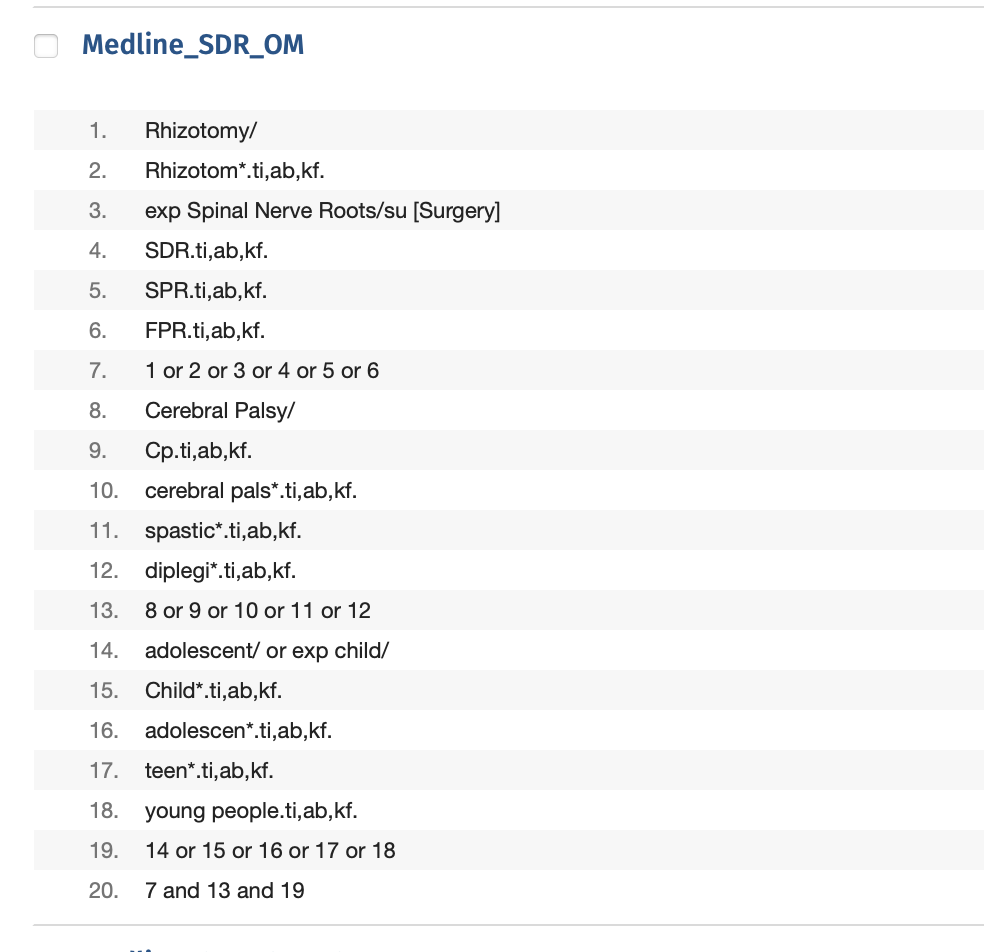


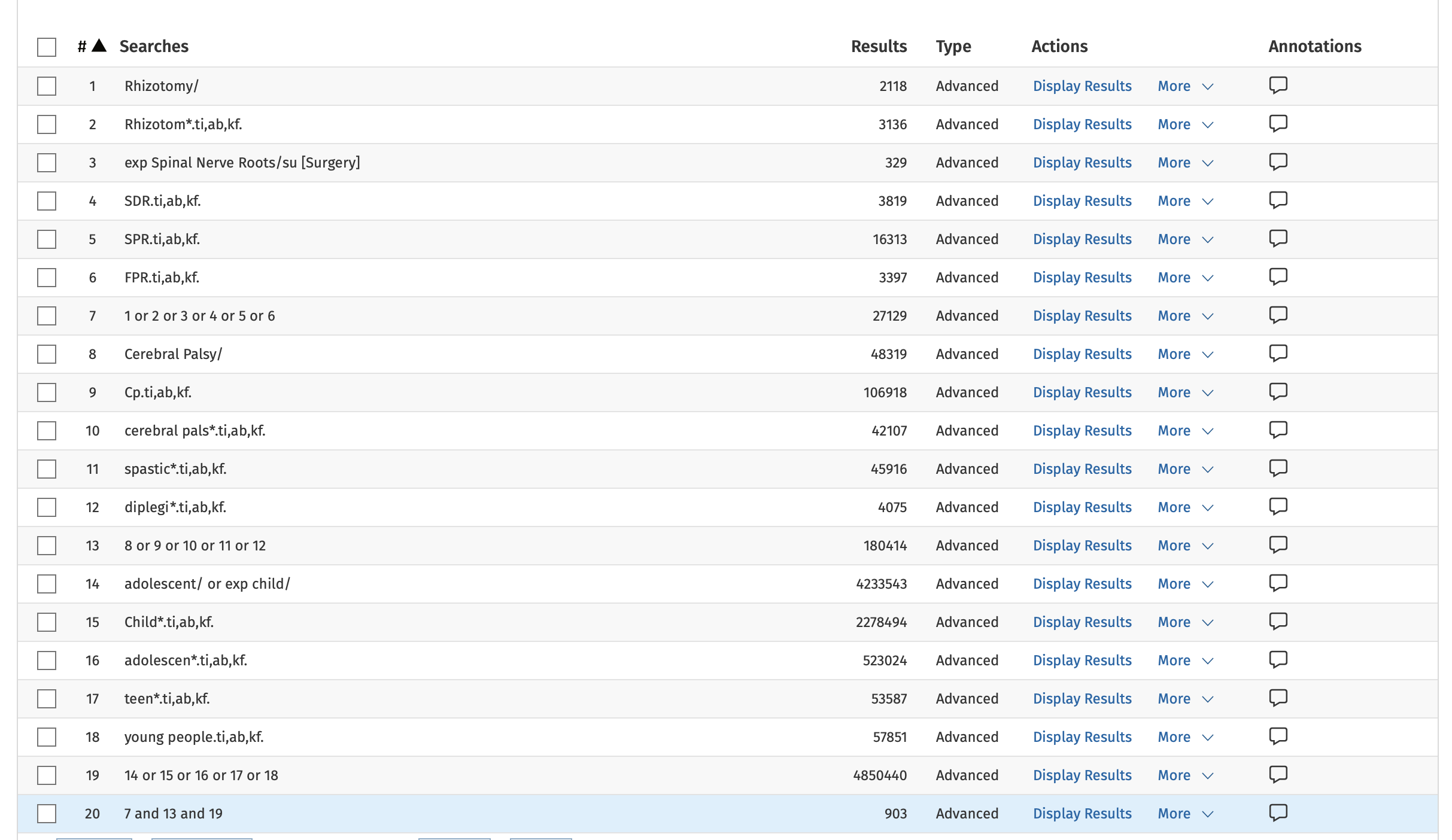


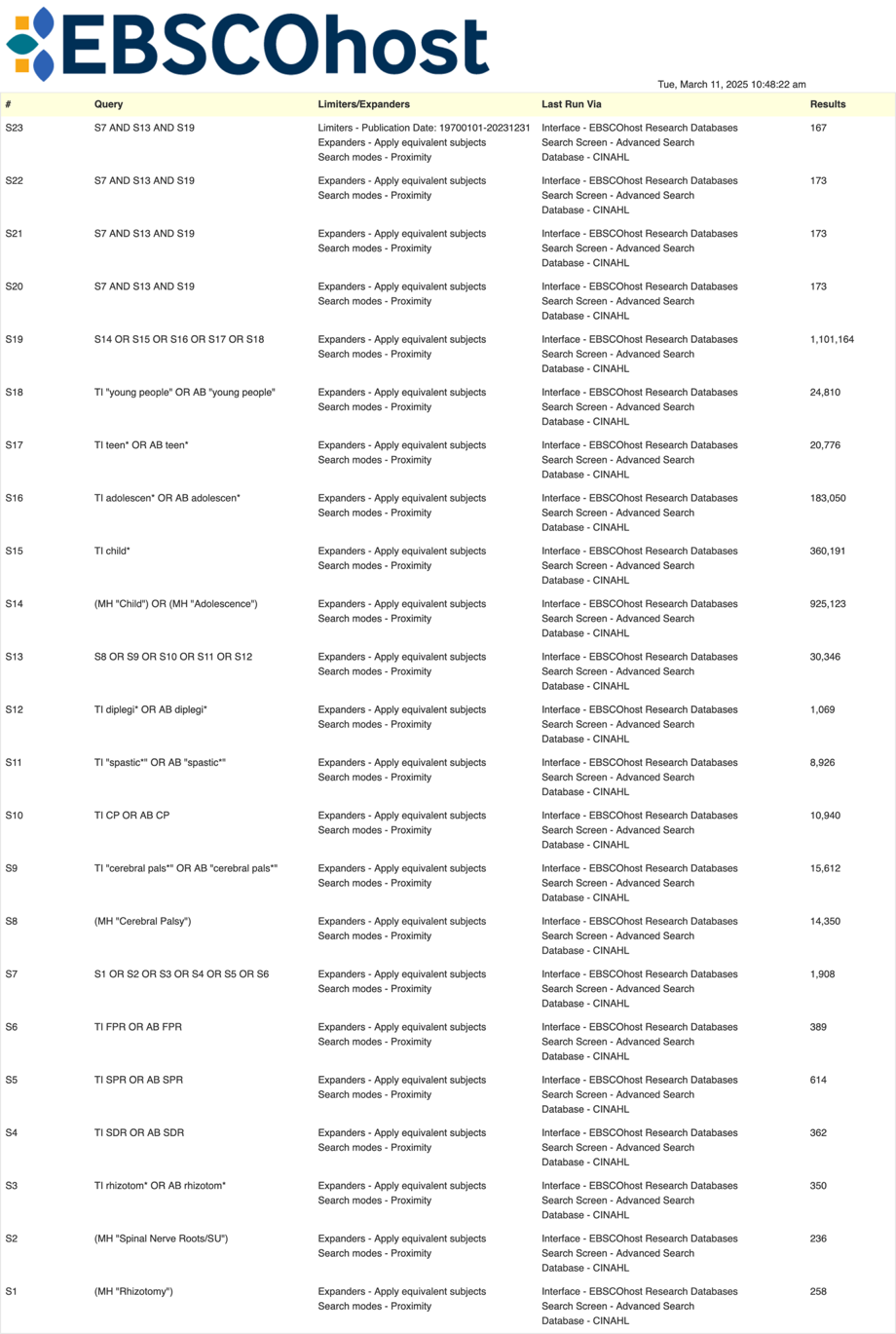
**CINHAL**
