# Supplementary material for: Outcomes of selective dorsal rhizotomy in ambulatory children and young people with cerebral palsy: A scoping review
Source: Dev Med Child Neurol. 2025 Sep 19;68(2):175–86. doi: 10.1111/dmcn.16496 (PMC12766555; doi:10.1111/dmcn.16496)
Supplement: Supplementary file 2 — Appendix S2: Data extraction instrument. [file DMCN-68-175-s002.docx]

### **Appendix S2: Data extraction instrument**

| **Scoping Review Details** | |
| --- | --- |
| Scoping review title: |  |
| Review objective(s): |  |
| Review question(s): |  |
| **Inclusion/Exclusion Criteria** | |
| Population | Bilateral spastic CP, GMFCS level I, II, III |
| Concept | SDR surgery (single or multilevel) |
| Context | All outcomes of SDR surgery |
| Types of evidence source | Published studies, all methodologies (except reviews, abstracts, commentaries, editorials) |

| **Evidence source details and characteristics** | |
| --- | --- |
| Citation details | e.g. author/s, date, title, journal, volume, issue, pages |
| Country | Study site, region, country |
| Context | Aim/purpose of study |
| Study design | Qualitative/ quantitative (RCT, Observational, Retrospective, Case-control, Survey, Case reports) |
| Control group | Yes/No |
| Level of evidence | I/II/III/IV/V (Oxford Centre for Evidence-based Medicine Scale) |
| Participants | GMFCS level, Topography, sample size, age at surgery, age at review/ follow-up range after surgery, sex (M:F); |
| **Details/Results extracted from source of evidence** | |
| ICF | Body Function & Structure/Activity/Participation/Environmental Factors/Personal Factors/ other (Quality of Life/ Level of satisfaction/ Goal-setting/ participants experience) |
| Outcome measure(s)/ evaluative tool(s) used |  |
| BS/BF – body structure/ body function | Spasticity/ ROM/Muscle strength/Gait parameters/pain/ SMC/ BMI/energy cost |
| Adverse events | Immediate post-op complications/ hip subluxation/ spinal deformity/ bladder/ sensory issues/ other |
| Activity | Gross motor function/ upper limb/ fine motor function/ functional mobility/ activities of daily living |
| Participation |  |
| Quality of life |  |
| Goals of intervention |  |
| Personal factors | Age, Sex, GMFCS level (functional level), comorbidities, |
| Environmental factors | Equipment- mobility aid, orthoses, access to services |
| Other | Satisfaction/ parental stress/ |
| Further interventions | Toxin, orthopaedic surgery, serial casting |
