# Supplementary material for: Outcomes of selective dorsal rhizotomy in ambulatory children and young people with cerebral palsy: A scoping review
Source: Dev Med Child Neurol. 2025 Sep 19;68(2):175–86. doi: 10.1111/dmcn.16496 (PMC12766555; doi:10.1111/dmcn.16496)

Appendix S3: Visual representation of data

Figure 1: Regional distribution of publications included in this scoping review

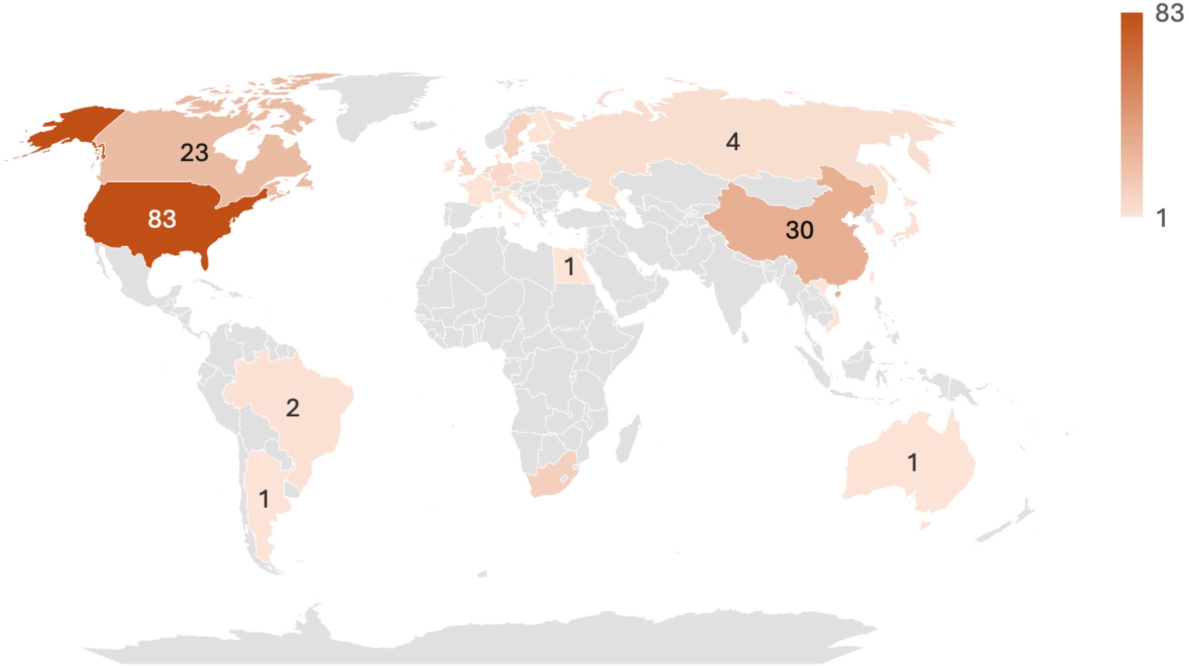

Country	No of publications
USA	83
China	30
Canada	23
South Africa	12
Sweden	10
The Netherlands	9
UK	8
Germany	7
Russia	4
Belgium	3
Taiwan	3
Czech Republic	3
Ireland	2
Brazil	2
Japan	2
South Korea	2
Italy	1
Mecico	1
Slovenia	1
France	1
Finland	1
Australia	1
Argentina	1
Egypt	1
Vietnam	1

Figure 2a: Bubble chart of all the outcomes measured: A proportionate representation of different outcomes, used more than once across different domains of the ICF. All the legends and data are included in the accompanying table. (*We will improve the quality of this figure for publication, as per the guidelines. For clarity, the figure has also been presented in 3 parts (2a, 2b and 2c)*)

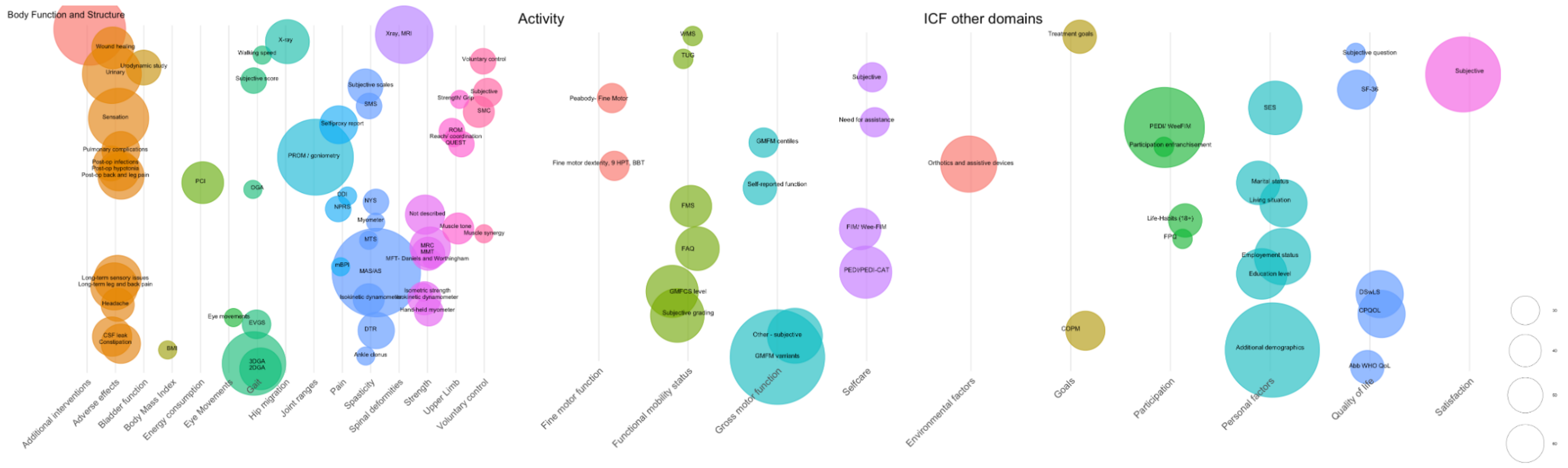

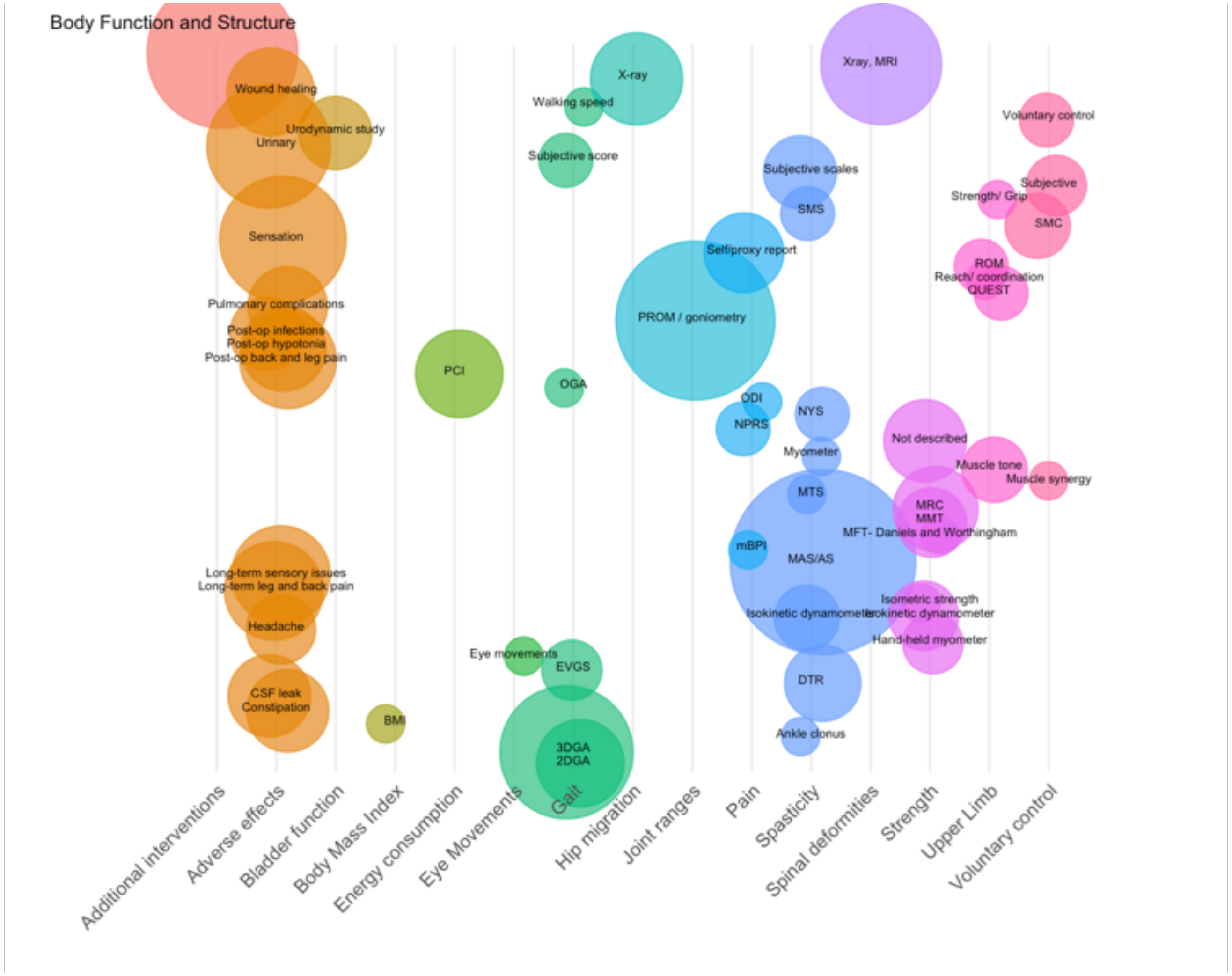

Figure 2a Body Function and Structure

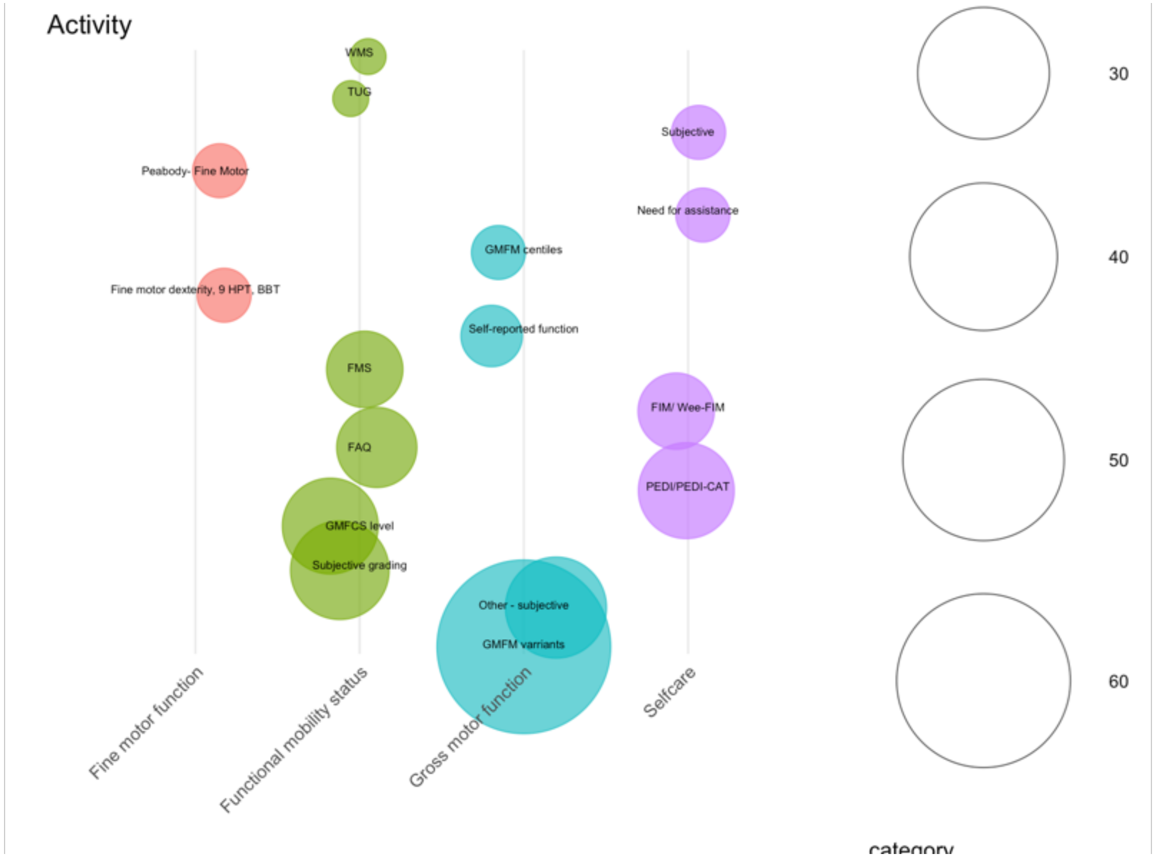

Figure 2b: Activity domain of the ICF

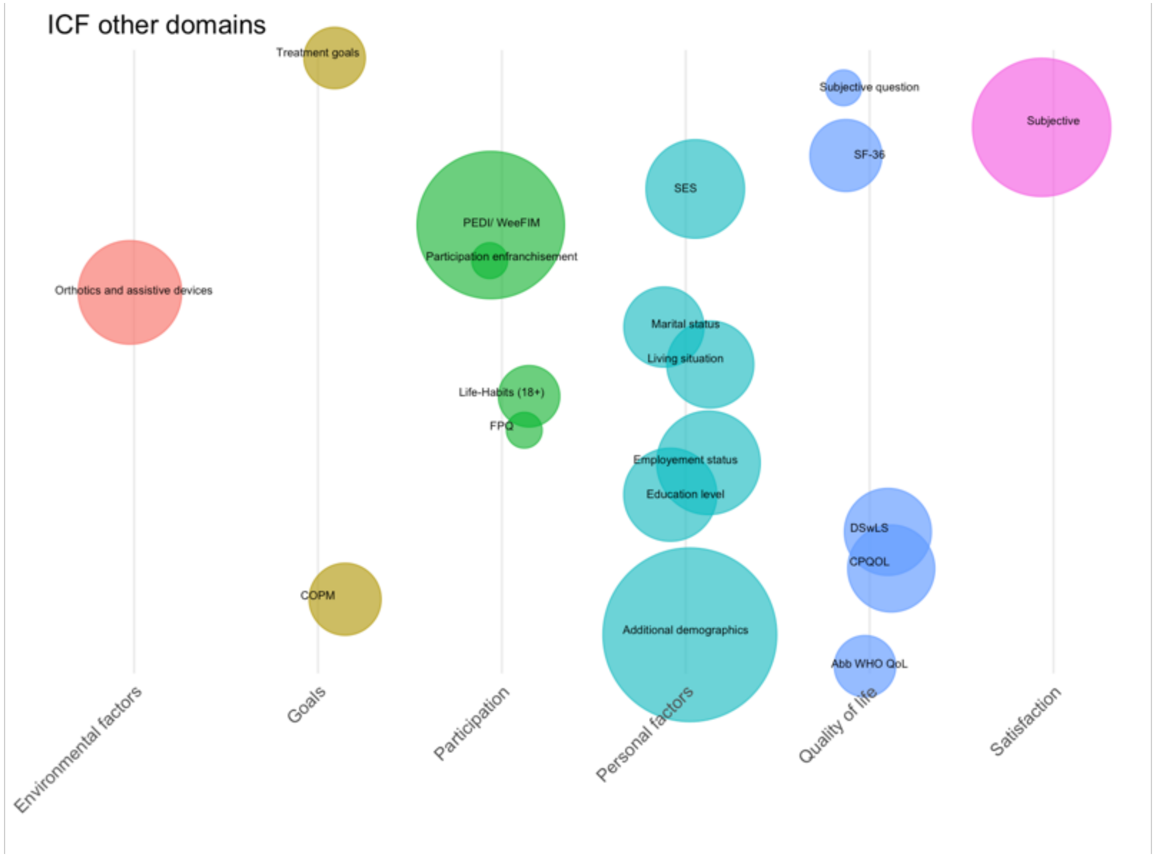

Figure 2c: All the other outcomes (Participation, Environmental and Personal factor, Goals, Quality of Life and Satisfaction.

Table 2: The information below includes the frequency (counts) of all the outcomes reported in the literature and is included here for reference.

domain_icf	category	tool	value
BFS	Spasticity	Modified Ashworth Scale/Ashworth scale (MAS/AS)	91
BFS	ROM	Passive range of movement (PROM) / goniometry	62
A	Gross Motor Function	Gross Motor Function Measure (GMFM) variants	60
BFS	Additional interventions	Additional interventions	54
BFS	Gait	3-Dimensional Gait Analysi (3DGA)	39
BFS	Adveffects	Abnormal sensation	34
BFS	Adveffects	urinary complications	32
BFS	Spinal Deformities	Spinal Deformities	30
PF	Pesonal factor	Additional demographics	30
P	Participation	PEDI/WeeFim	20
BFS	Adveffects	Long-term leg and back pain	17
BFS	Adveffects	long-term lsensory issues	17
Satisfaction	Satisfaction of procedure	Subjective	17
BFS	Adveffects	Post-op back and leg pain	16
A	Gross Motor Function	Other-subjective	15
BFS	Hip migration	X-ray	14
A	Functional Mobility/ Ambualtory Status	Subjective grading	14
A	Functional Mobility/ Ambualtory Status	GMFCS level	13
A	ADLs/ Selfcare	Pediatric Evaluation of Disability Inventory (Pedi/Pedi-Cat)	13
BFS	Gait	2-Dimensional Gait Analysi (2DGA)	12
BFS	Energy consumption	Physiological Cost Index	12
BFS	Adveffects	Wound healing	12
BFS	Ms Strength	Medical Research Council (MRC)	11
BFS	Adveffects	Post-operative hypotonia	11
BFS	Ms Strength	Not described	10
BFS	Adveffects	Constipation	10
BFS	Adveffects	CSF leak	10
BFS	Pain	Participant/ parent reported -pain	9
BFS	Adveffects	Pulmonary Complications	9
BFS	Spasticity	Deep Tendon Reflexes (DTR)	8
A	Functional Mobility/ Ambualtory Status	Functional Assesment Questionnaire (FAQ)	8
PF	Pesonal factor	Employement Status	8
EF	Environmental factor	Orthotics and assistive devices	8
BFS	Spasticity	Subjective scale	7
BFS	Bladder Function	Urodynamic study	7
BFS	Adveffects	Post-operative infections	7
A	Functional Mobility/ Ambualtory Status	Functional Mobility Scale (FMS)	7
A	ADLs/ Selfcare	Functional Independence Measure (FIM/ Wee-FIM)	7
PF	Pesonal factor	Socioeconomic status (SES)	7
BFS	Ms Strength	Manual Muscle Testing (MMT)	6
BFS	Ms Strength	Isokinetic Dynamometer	6
BFS	Adveffects	Headache	6
PF	Pesonal factor	Education level	6
BFS	Spasticity	Isokinetic dynamometer	5
BFS	Voluntary control	Selective Motor Control (SMC)	5
BFS	Upper Limb/ Fine Motor Function	Muscle tone	5
BFS	Upper Limb/ Fine Motor Function	Muscle tone	5
PF	Pesonal factor	Living situation	5
QoL	QoL	Cerebral Palsy Quality of Life (CPQOL)	5
QoL	QoL	Diener Satisfaction with Life Scale (DSwLS)	5
BFS	Ms Strength	Hand-held myometer	4
BFS	Gait	Edinburgh Visual Gait Scale (EVGS)	4
BFS	Voluntary control	Subjective grading	4
A	Gross Motor Function	Self-reported functional measure (SRFM)	4
PF	Pesonal factor	Marital status	4
BFS	Spasticity	New York Scale	3
BFS	Spasticity	Spastidty Measurement System (SMS)	3
BFS	Gait	Subjective score	3
BFS	Voluntary control	Voluntary control	3
BFS	Pain	Numeric pain rating scale (NPRS)	3
BFS	Upper Limb/ Fine Motor Function	Range of Motion (ROM)	3
BFS	Upper Limb/ Fine Motor Function	Quality of Upper Extremities Skills Test (QUEST)	3
A	Gross Motor Function	GMFM centiles	3
A	ADLs/ Selfcare	Subjective	3
A	ADLs/ Selfcare	Need for assistance	3
A	Upper Limb/ Fine Motor Function	Peabody_Fine Motor	3
A	Upper Limb/ Fine Motor Function	Fine Motor dexterity, 9 hole peg test, box and block	3
QoL	QoL	SF-36	3
Goals	Goal Setting	Canadian Occupational Performance Measure (COPM)	3
BFS	Spasticity	Myometer	2
BFS	Spasticity	Modified Tardieu scale (MTS)	2
BFS	Spasticity	Ankle Clonus	2
BFS	Ms Strength	Isometric strength	2
BFS	Ms Strength	Manual Function Test (MFT) - Daniels and Worthingham	2
BFS	Gait	Observational Gait-Analysis (OGA)	2
BFS	Gait	Walking speed	2
BFS	BMI	BMI centiles and z scores	2
BFS	Voluntary control	Muscle Synergy	2
BFS	Pain	Modified Brief Pain Inventory (mBPI)	2
BFS	Pain	Oswestry Disability Index (ODI)	2
BFS	Upper Limb/ Fine Motor Function	Strength/ Grip	2
BFS	Upper Limb/ Fine Motor Function	Reach/ coordination	2
BFS	Eye Movements	Eye Movements	2
A	Functional Mobility/ Ambualtory Status	Wilson Mobility Scale	2
A	Functional Mobility/ Ambualtory Status	Timed Up and Go (TUG)	2
P	Participation	Social participation - Life-Habits (18+)	2
QoL	QoL	Abb WHO QoL	2
Goals	Goal Setting	Description of treatment goals	2
BFS	Spasticity	Dynamic electromyography (EMG)	1
BFS	Spasticity	H-reflex	1
BFS	Spasticity	Hyperactivity Assessment Tool (HAT)	1
BFS	Spasticity	Barry-Albright Dystonia scale (BADS: Mixed spasticity and dystonia)	1
BFS	Spasticity	Weighted average of 5 measures of spasticity	1
BFS	Spasticity	Repeated movement test	1
[illegible]	[illegible]	[illegible]	[illegible]
A	Functional Mobility/ Ambualtory Status	Timed Up and Go (TUG)	2
P	Participation	Social participation - Life-Habits (18+)	2
QoL	QoL	Abb WHO QoL	2
Goals	Goal Setting	Description of treatment goals	2
BFS	Spasticity	Dynamic electromyography (EMG)	1
BFS	Spasticity	H-reflex	1
BFS	Spasticity	Hyperactivity Assessment Tool (HAT)	1
BFS	Spasticity	Barry-Albright Dystonia scale (BADS: Mixed spasticity and dystonia)	1
BFS	Spasticity	Weighted average of 5 measures of spasticity	1
BFS	Spasticity	Repeated movement test	1
BFS	Spasticity	Graded on EMG response to manual stretch	1
BFS	Spasticity	Not described	1
BFS	ROM	Digital goniometer	1
BFS	ROM	Video system	1
BFS	ROM	Subjective grading	1
BFS	Ms Strength	Lovett 6 grade scoring method	1
BFS	Ms Strength	Abbott grading	1
BFS	Balance	Ground Reaction Force (GRF)	1
BFS	Balance	Balance system in sitting	1
BFS	Voluntary control	Selective control assessment of lower extremity (SCALE)	1
BFS	Quality of Movement	Subjective video analysis	1
BFS	Quality of Movement	Gross Motor Performance Measure (GMPM)	1
BFS	Quality of Movement	Quality Function Measure (QFM)	1
BFS	Pain	Two domains of Multiple Sclerosis spasticity Scale (MS SS)	1
BFS	Pain	Patient Reported Outcome Measurement Information System (PROMIS)	1
BFS	Pain	Fatigue Severity Scale (FSS)	1
BFS	Pain	Observational scale of behavioural distress (OSBD)	1
BFS	Pain	Observer rating of pain and anxiety (ORPA)	1
BFS	Pain	Graphic rating scale of anxiety and pain (GRSAP)	1
BFS	Pain	Faces-Legs-Activity-Cry-Consolability (FLAAC)	1
BFS	Balance	Pediatric Bergs Balance Scale (PBS)	1
BFS	Phonation	Phonation	1
BFS	Cognitive performance	Visual Attention Task	1
BFS	Cognitive performance	Wood-Stock Johnson Psychological Battery	1
BFS	Adveffects	Fatigue	1
BFS	Adveffects	Sudden fall	1
BFS	Adveffects	Spinal cord tethering	1
A	Gross Motor Function	Bayley Scales of Infant Development (BSID)	1
A	Functional Mobility/ Ambualtory Status	Peacock grading	1
A	Functional Mobility/ Ambualtory Status	Illinois-St Louis scale	1
A	Exercise Tolerance	6-Minute Walk Test (6MWT)	1
A	Exercise Tolerance	1-Minute Walk Test (1MWT)	1
A	ADLs/ Selfcare	RIC-FAS	1
A	Level of physical activity	Saltin-Grimbly Scale	1
A	Upper Limb/ Fine Motor Function	Bruininks-Oseretsky Test of Motor Proficiency (BOTMP)	1
P	Participation	Participation enfranchisement	1
P	Participation	Frequency of participation questionnaire	1
QoL	QoL	Subjective question	1

Sample size (number of participants) across studies:

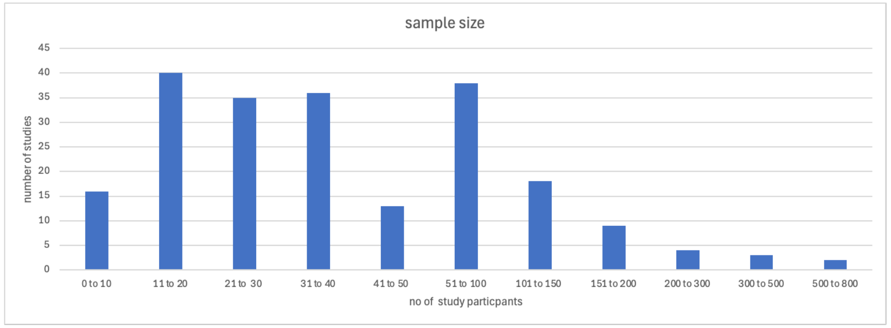

Types of study design:

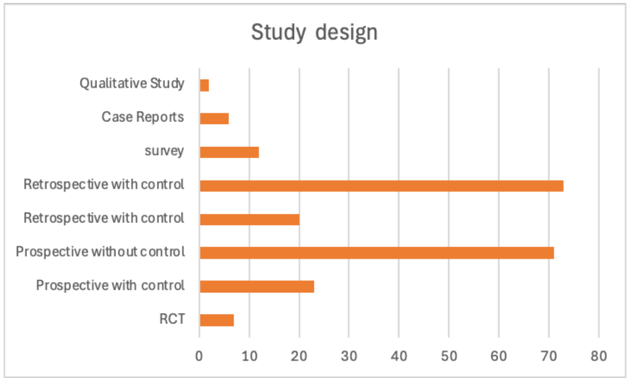

Type of outcome measures used:

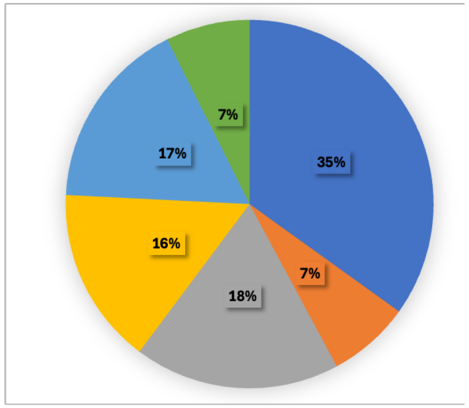

Percentage distribution of outcomes across ICF domains for different lengths of follow-up:

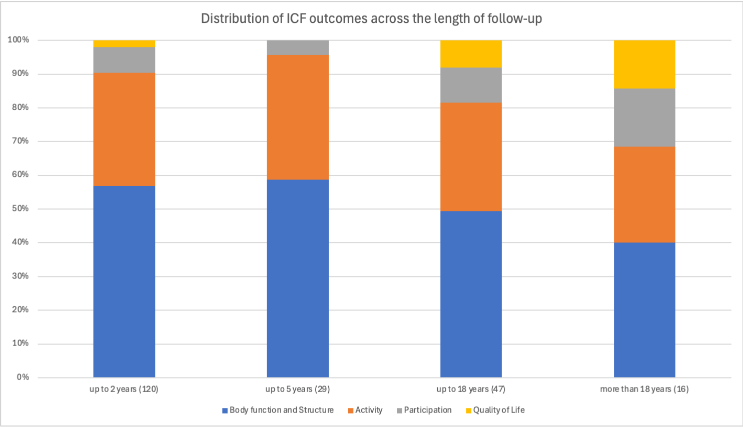

Supplement: Supplementary file 3 — Appendix S3: Visual representation of data. [file DMCN-68-175-s004.pdf]
