# Supplementary material for: Outcomes of selective dorsal rhizotomy in ambulatory children and young people with cerebral palsy: A scoping review
Source: Dev Med Child Neurol. 2025 Sep 19;68(2):175–86. doi: 10.1111/dmcn.16496 (PMC12766555; doi:10.1111/dmcn.16496)
Supplement: Supplementary file 4 — Appendix S4: Outcome domains and outcome measures used in the literature and ICF coding. [file DMCN-68-175-s001.docx]

**Appendix S4**

**Outcome domains and outcome measures used in the literature and ICF coding**

|  | | | |  |
| --- | --- | --- | --- | --- |
| **Outcome domain** | **ICF code** | **Measurement tool** | **N** | **Study ID** |
| Body Function and Structure | |  |  |  |
| Spasticity | b735—muscle tone functions | Modified Ashworth Scale/Ashworth scale | 91 | 6, 8, 16, 17, 18, 19, 21, 23, 27, 34, 35, 36, 38, 39, 40, 51, 52, 53, 54, 55, 56, 63, 65, 66, 67, 79, 80,73, 75, 76, 78, 84, 85 86, 87  88, 89, 94, 98, 99, 105, 117, 108, 109, 115, 124, 123, 126, 127, 130, 141, 148, 149, 150,  156, 157, 158, 159, 163, 164 165, 166, 168, 172, 169, 170, 167, 176, 178, 179, 182, 181, 180, 183, 184, 185, 190, 191, 195, 197, 198, 199, 203, 204, 205, 207, 208, 209, 210, 211, 213 |
|  |  | Isokinetic dynamometer | 5 | 41,42,44,45,46 |
|  |  | Deep tendon reflexes | 8 | 22, 66, 78, 86, 102, 114, 115, 203 |
|  |  | Myometer | 2 | 166, 171 |
|  |  | New York University Tone scale | 3 | 1, 118, 122 |
|  |  | Dynamic electromyography | 1 | 7 |
|  |  | Modified Tardieu scale | 2 | 103, 189 |
|  |  | Ankle clonus | 2 | 102, 141 |
|  |  | H-reflex | 1 | 183 |
|  |  | Hypertonia Assessment Tool | 1 | 55 |
|  |  | Spasticity measurement system | 3 | 65, 114, 161 |
|  |  | Other subjective (mild/moderate/severe) | 7 | 22, 70, 95, 97, 146, 174, 206 |
|  |  | Barry-Albright Dystonia scale | 1 | 55 |
|  |  | Weighted average of 5 measures of spasticity | 1 | 107 |
|  |  | Repeated movement test | 1 | 190 |
|  |  | Graded on EMG response to manual stretch | 1 | 5 |
|  |  | Not described | 1 | 147 |
| Joint ranges | b710 – mobility of joint functions | Joint ranges of movements -goniometry | 62 | 1, 5, 6, 7, 13, 16, 17, 18, 19, 21, 27, 35, 39, 53, 56, 63, 67, 70, 81, 83, 85, 88, 89, 98, 99,103, 107, 110, 111, 112, 114, 115, 118, 122, 126, 127, 129, 130, 134, 148, 149, 150, 158, 163, 164, 165, 166, 171, 168, 172, 169, 170, 176, 178, 179, 181, 180, 183, 191, 197, 203, 210 |
|  |  | Digital gonio | 1 | 153 |
|  |  | Video system | 1 | 43 |
|  |  | Subjective grading | 1 | 87 |
| Muscle strength | b730—muscle power function | Medical Research Council scale for muscle strength | 11 | 6, 18, 27, 63, 84, 94, 112, 159, 165, 172, 167 |
|  |  | Manual Muscle testing | 6 | 17, 31, 127, 176, 209, 210 |
|  |  | Isokinetic dynamometer | 6 | 41, 42, 44, 45, 46, 154, |
|  |  | Isometric strength | 2 | 15, 88 |
|  |  | Hand-held myometer | 4 | 171, 169,170, 191 |
|  |  | Daniels and Worthingham’s muscle testing | 2 | 8, 51 |
|  |  | Lovett 6-grade scoring method | 1 | 78 |
|  |  | Abbott grading | 1 | 195 |
|  |  | Not described | 10 | 56, 95, 89, 107, 108, 147, 146, 148,164, 213 |
| Gait analysis | b770—gait pattern functions | Three-Dimensional Gait Analysis | 39 | 3, 4, 7, 8, 13, 18, 31, 39, 41, 42, 47, 52, 53, 59, 80, 81, 84, 91, 92, 107, 117,  109, 110, 112, 114, 116, 127, 134, 150, 155, 156,160, 162, 176, 182, 181, 187, 203, |
|  |  | Two-Dimensional ~Gait Analysis | 12 | 61, 81, 88, 89, 96, 165, 166, 175, 180, 201, 202, 205 |
|  |  | Edinburgh Visual Gait Scale | 4 | 61, 137, 153, 190 |
|  |  | Observational Gait Analysis | 2 | 21, 67, |
|  |  | Walking speed | 2 | 209, 185 |
|  |  | Subjective scores | 3 | 25, 111, 129 |
| Balance | b755 -involuntary movement reaction functions | Ground Reaction Force, using force plates | 1 | 155 |
|  |  | Balance system in sitting | 1 | 207 |
|  |  | Paediatric Bergs Balance | 1 | 2 |
| Energy consumption | B455- exercise tolerance function | Energy consumption during walk test Physiological Cost Index | 12 | 2, 18, 64, 67, 107, 127,  160, 169, 170, 180, 185 209 |
| Body Mass Index | b530 Weight maintenance | BMI centiles and z scores | 2 | 64, 200 |
| Selective Motor Control | b760—control of voluntary movement functions | Subjective grading | 4 | 25, 95, 127, 191 |
|  |  | Selective Control Assessment of the Lower Extremity | 1 | 18 |
|  |  | Muscle Synergy | 2 | 137, 201 |
|  |  | Voluntary control | 3 | 70, 135, 169 |
|  |  | Selective Motor Control | 5 | 21, 109, 150, 165, 176 |
| Quality of movement | B7602- coordination of voluntary movements | Subjective video analysis of posture and transitions | 1 | 122 |
|  |  | Gross Motor Performance Measure | 1 | 14 |
|  |  | Quality Function Measure | 1 | 29 |
| Pain | b280- sensation of pain | Numeric Pain Rating Scale | 3 | 74,144, 142, |
|  |  | Modified Brief Pain Inventory | 2 | 127, 178 |
|  |  | Oswestry Disability Index | 2 | 94, 192, |
|  |  | Two domains of Multiple Sclerosis spasticity Scale | 1 | 107 |
|  |  | Participant/ parent-reported pain | 9 | 30, 67, 74, 81, 94, 105, 138, 164, 192, |
|  |  | PROMIS | 1 | 33 |
|  |  | Fatigue Severity Scale | 1 | 33 |
|  |  | Observational scale of behavioural distress | 1 | 118 |
|  |  | Observer rating of pain and anxiety | 1 | 118 |
|  |  | Graphic rating scale of anxiety and pain | 1 | 118 |
|  |  |  |  |  |

| **Outcome domain** | **ICF code** | **Measurement tool** | **N** | **Study ID** |
| --- | --- | --- | --- | --- |
| Activity and Participation | |  |  |  |
| Gross motor function | d420—transferring oneself/d469—walking and moving, other specified and unspecified | Gross Motor Function Measure- 88/66 | 60 | 2, 4, 6, 8, 12, 14, 18, 21, 29, 35, 38, 39, 41, 42, 47, 48, 51, 55, 56, 60, 62, 65, 67, 70, 76, 79, 80, 83, 107, 117, 114, 115, 122, 124, 126, 148, 149, 131, 130, 156, 157, 159, 163, 165, 172, 169, 170, 167, 176, 178, 179, 184,189, 190, 197, 199, 203, 204, 210 |
|  |  | Gross Motor Function Measure- Centiles | 3 | 58, 117, 176, |
|  |  | Other variants/ non-validated subjective scales /Peacock grading system | 16 | 9, 16, 63, 75, 86, 88, 89, 95, 102, 108, 147, 146, 166, 183, 205, 211 |
|  |  | Bayley’s Scale of Infant Development, | 1 | 118 |
|  |  | Self-Reported | 4 | 33, 138, 141, 171 |
| Functional mobility Status | d465 Moving around using equipment (wheelchair, skates, etc.) | Functional Mobility Scale | 7 | 56, 93, 107, 127, 165, 178, 193, |
|  | d460 – moving around in different locations | Functional Assessment Questionnaire | 8 | 27, 39, 55, 64, 107, 117, 127, 160,185 |
|  |  | Gross Motor Functional Classification System | 13 | 6, 31, 39, 74, 85, 91, 117,144, 149, 158, 159, 199, 210 |
|  |  | Wilson Mobility Scale | 2 | 178, 179 |
|  |  | Subjective Ambulatory Status | 14 | 63, 66, 70, 86, 97, 111, 115, 129, 133, 132, 138, 168, 170, 206 |
|  |  | Illinois-St Louis scale | 1 | 75 |
|  |  | Timed Up and Go | 2 | 55, 191 |
|  | b455—exercise tolerance | 6 – Minute Walk Test | 1 | 55 |
|  |  | 1 – Minute Walk Test | 1 | 203 |
| ADLs | multiple domains: d510,d520, d530, d540, d420, d450, d455, d460, d470, d750, d920, e310, e320, e355, e115, e120 | Pediatric Evaluation of Disability Inventory (PEDI), PEDI-Computer Assisted Test | 13 | 11, 14, 21, 37, 38, 49, 55, 67, 82, 121, 131, 190, |
|  |  | Wee Functional Independence Measure/ FIM | 7 | 18, 104, 128, 129, 167, 178, 183, |
|  |  | Subjective/ ordinal scale | 3 | 68, 170, 182, |
|  |  | Rehab Institute of Chicago Functional Assessment Scale | 1 | 99 |
| Anxiety and depression | d240 handling stress and other psychological demands | The Hospital Anxiety and Depression Scale (HADS) is a questionnaire | 1 | 191 |
| Physical activity | d920 physical activity for recreation and leisure | Saltin-Grimbly Scale of physical activity | 1 | 178 |
| Participation | Multiple ICF domains | Participation enfranchisement | 1 | 107 |
|  |  | Social participation - Life-Habits (18+) | 2 | 93, 187 |
|  |  | Frequency of participation questionnaire | 1 | 127 |
|  |  | Subjective question | 1 |  |

**Quality of Life**

| **Measurement tool** | **N** | **Study reference number** |
| --- | --- | --- |
| CPQOL | 5 | 27, 55, 100, 152, 176 |
| Abbreviated WHO QoL | 2 | 107,127 |
| Diener Satisfaction with Life | 5 | 74, 107, 127, 144, 142 |
| Subjective question | 2 | 74, 158 |
| SF-36 | 3 | 139, 178, 191 |

**Goals and Satisfaction**

|  | **Measurement tool** | **N** | **Study reference number** |
| --- | --- | --- | --- |
| Goals | COPM | 3 | 21,67, 100 |
| Satisfaction of procedure |  | 17 | 1,27, 70, 74, 85, 93, 96, 133, 132, 144, 140, 138, 139, 142, 147, 146, 189 |

**Other Outcomes**

| **Outcome** | **ICF code** | **Measurement tool** | **N** | **Study reference number** |
| --- | --- | --- | --- | --- |
| **Bladder function** | b620 Urination functions | Urodynamic study | 7 | 21, 26, 67, 72, 88, 89, 177 |
| **UL/ Fine motor function (n=13)** | d445—hand and arm use | Upper limb reach, coordination and hand function | 2 | 9, 37 |
|  |  | Upper limb muscle tone | 5 | 17, 21, 54, 98, 99 |
|  |  | MACS level | 3 | 74, 144, 142 |
|  |  | ROM | 3 | 17, 98, 99 |
|  |  | strength-MMT, grip/grasp | 2 | 49, 17 |
|  |  | QUEST- | 3 | 104, 125, 167 |
|  |  | Peabody -Fine motor | 3 | 17, 120, 170 |
|  | d440 Fine hand use (picking up, grasping) | Fine motor Dexterity, Reach, grasp, hand Function, 9 HPT, box & block | 3 | 17, 49, 99 |
|  |  | BOTMP | 1 | 17 |
| **Eye movements** | B210 |  | 2 | 71, 73 |
| **Phonation** |  |  | 1 | 73 |
| **Cognitive performance** | B117 | Visual attention task | 1 | 32 |
|  |  | Wood-stock Johnson Psychological battery | 1 | 32 |

**Adverse effects and complications**

| **Complication/ adverse event** | **N** | **Study reference number** |
| --- | --- | --- |
| **Abnormal sensation in the first 6 weeks** | 34 | 10, 16, 23, 31, 34, 39, 67, 70, 77, 78, 88, 89,114, 115,130,140, 139, 146, 147, 148, 149, 157, 163, 166, 171, 173, 176, 179, 185, 190, 194, 205, 206, 210 |
| **Urinary complications** | 32 | 10, 16, 21, 23, 26, 31, 39, 67, 70, 78, 88, 89, 95, 115, 122, 121, 120, 124, 130,144, 142, 157, 171, 170, 173, 176,177, 179, 183, 193, 206 |
| **Post-op back and leg pain** | 16 | 10, 23, 30, 50, 66, 77, 88, 114, 130, 158, 173, 174, 179,163, 194, 214 |
| **Long-term leg and back pain** | 17 | 50, 77, 81, 67, 74, 88, 93, 94, 95, 105, 107, 130,144, 140, 142, 158, 164 |
| **constipation** | 10 | 10, 23, 77, 124, 130, 173, 176, 177, 185, 188 |
| **Long-term sensory issues** | 9 | 39, 60, 115, 144, 142, 147, 146, 176,179 |
| **Wound healing** | 12 | 23, 36, 39, 70, 147, 149, 157, 158, 176, 185, 194 |
| **CSF leak** | 10 | 23, 35, 39, 78, 130, 139, 147, 158, 173, 194, 210, |
| **Pulmonary complications** | 9 | 77, 88, 89,124, 130, 173, 183, 188, 194 |
| **Post-operative infections** | 7 | 10, 39, 77, 171, 183, 194, 203 |
| **Post-operative hypotonia and weakness** | 11 | 88, 89, 114, 158, 163, 166,176,179, 184, 185, 194 |
| **headaches** | 6 | 23, 39, 77, 173, 185, 206 |
| **Sudden falls** | 1 | 60 |
| **Spinal cord tethering** | 1 | 10 |

**Studies reporting incidence of orthopaedic interventions, spinal deformities and hip migration:**

|  | **N** | **Study reference number** |
| --- | --- | --- |
| **Orthopaedic interventions** | 54 | 6, 12, 19, 20, 24, 33, 38, 52, 55, 56, 63, 66,70, 74, 75, 83, 85, 89, 91, 92, 94, 96, 102, 103, 106, 107, 110, 111, 112, 116, 122, 121, 127, 130, 133, 132, 134, 137, 144, 138, 142, 147, 146, 153, 164, 172, 175, 178, 179, 184, 189, 191, 210, 211 |
| **Incidence of spinal deformities** | 30 | 10, 12, 21, 23, 35, 50, 57, 58, 67, 75, 78, 81, 89, 90, 94, 101, 105, 130, 144, 142, 147, 146, 145, 151, 168, 186, 189, 192, 198, 208 |
| **Hip migration** | 14 | 19, 20, 21, 48, 67, 68, 69, 75, 89, 114, 119, 130, 143, 189, |

**Studies reporting contextual factors:**

|  |  | **N** | **Study reference number** |
| --- | --- | --- | --- |
| **Personal factors** | Additional demographic characteristics of participants (e.g. birth history, comorbidities and cognitive level) | 30 | 40, 69, 82, 83, 90, 92, 93, 95, 99, 100, 105, 114, 115, 118, 131, 130, 147, 146, 151, 162, 168, 176, 185, 188, 191, 194, 213, 214, 210 |
|  | Employment status | 8 | 33, 74, 92, 93*, 95*, 114, 178, 193 |
|  | Socioeconomic status | 7 | 91, 93, 95, 114, 193, 191, 192 |
|  | Education level | 6 | 33, 74, 92, 93, 178, 193 |
|  | Living situation | 5 | 33, 74, 93, 178, 193, |
|  | Marital status | 4 | 92, 93, 114, 193 |
| **Environmental factors** | Orthotics and assistive devices | 8 | 2, 5, 15, 59, 75, 92, 103, 164 |

**Reference (all studies included in this review)**

1. Abbott R, Johann-Murphy M, Shiminski-Maher T, Quartermain D, Forem SL, Gold JT, et al. Selective dorsal rhizotomy: Outcome and complications in treating spastic cerebral palsy. Neurosurgery. 1993;33(5):851-7.

2. Abd-Elmonem AM, Ali HA, Saad-Eldien SS, Rabiee A, Abd El-Nabie WA. Effect of physical training on motor function of ambulant children with diplegia after selective dorsal rhizotomy: A randomized controlled study. Neurorehabilitation. 2023;53(4):547-56.

3. Abel MF, Damiano DL, Gilgannon M, Carmines D, Kang HG, Bennett BC, et al. Biomechanical changes in gait following selective dorsal rhizotomy. Journal of Neurosurgery. 2005;102(2):157-62.

4. Abel MF, Damiano DL, McLaughlin JF, Song KM, Graubert CS, Bjornson KF. Comparison of functional outcomes from orthopedic and neurosurgical interventions in spastic diplegia. Neurosurgical focus. 1998;4(1):e2.

5. Adams JM, Cahan LD, Perry J, Beeler LM. Foot contact pattern following selective dorsal rhizotomy. Pediatric Neurosurgery. 1995;23(2):76-81.

6. Ailon T, Beauchamp R, Miller S, Mortenson P, Kerr JM, Hengel AR, et al. Long-term outcome after selective dorsal rhizotomy in children with spastic cerebral palsy. Childs Nervous System. 2015;31(3):415-23.

7. Ates F, Brandenburg JE, Kaufman KR. Effects of Selective Dorsal Rhizotomy on Ankle Joint Function in Patients With Cerebral Palsy. Frontiers in Pediatrics. 2020;8.

8. Bakir MS, Gruschke F, Taylor WR, Haberl EJ, Sharankou I, Perka C, et al. Temporal but Not Spatial Variability during Gait Is Reduced after Selective Dorsal Rhizotomy in Children with Cerebral Palsy. Plos One. 2013;8(7).

9. Beck AJ, Gaskill SJ, Marlin AE. IMPROVEMENT IN UPPER EXTREMITY FUNCTION AND TRUNK CONTROL AFTER SELECTIVE POSTERIOR RHIZOTOMY. American Journal of Occupational Therapy. 1993;47(8):704-7.

10. Belanger K, McKay W, Oleszek J, Graber S, Wilkinson C. Spinal cord tethering after selective dorsal rhizotomy below the conus medullaris. Child's Nervous System. 2022;38(11):2129-32.

11. Bloom KK, Nazar GB. Functional assessment following selective posterior rhizotomy in spastic cerebral palsy. Child's Nervous System. 1994;10(2):84-6.

12. Bolster EAM, Van Schie PEM, Becher JG, Van Ouwerkerk WJR, Strijers RLM, Vermeulen RJ. Long-term effect of selective dorsal rhizotomy on gross motor function in ambulant children with spastic bilateral cerebral palsy, compared with reference centiles. Developmental Medicine and Child Neurology. 2013;55(7):610-6.

13. Boscarino LF, Ounpuu PTS, Davis Iii RB, Gage JR, DeLuca PA. Effects of selective dorsal rhizotomy on gait in children with cerebral palsy. Journal of Pediatric Orthopaedics. 1993;13(2):174-9.

14. Buckon CE, Thomas SS, Piatt Jr JH, Aiona MD, Sussman MD. Selective dorsal rhizotomy versus orthopedic surgery: A multidimensional assessment of outcome efficacy. Archives of Physical Medicine and Rehabilitation. 2004;85(3):457-65.

15. Buckon CE, Thomas SS, Harris GE, Piatt JH, Aiona MD, Sussman MD. Objective measurement of muscle strength in children with spastic diplegia after selective dorsal rhizotomy. Archives of Physical Medicine and Rehabilitation. 2002;83(4):454-60.

16. Buckon CE, Thomas SS, Pierce R, Piatt Jr JH, Aiona MD. Developmental skills of children with spastic diplegia: Functional and qualitative changes after selective dorsal rhizotomy. Archives of Physical Medicine and Rehabilitation. 1997;78(9):946-51.

17. Buckon CE, Sienko Thomas S, Aiona MD, Piatt JH. Assessment of upper-extremity function in children with spastic diplegia before and after selective dorsal rhizotomy. Developmental medicine and child neurology. 1996;38(11):967-75.

18. Carraro E, Zeme S, Ticcinelli V, Massaroni C, Santin M, Peretta P, et al. Multidimensional outcome measure of selective dorsal rhizotomy in spastic cerebral palsy. European Journal of Paediatric Neurology. 2014;18(6):704-13.

19. Carroll KL, Moore KR, Stevens PM. Orthopedic procedures after rhizotomy. Journal of Pediatric Orthopaedics. 1998;18(1):69-74.

20. Chan WM, Choi KYA, Sun KW, Fong D, Yam KY. Hip Development After Selective Dorsal Rhizotomy in Patients with Cerebral Palsy. Journal of Orthopaedics, Trauma and Rehabilitation. 2013;17(2):82-6.

21. Chan SHS, Yam KY, Yiu-Lau BPH, Poon CYC, Chan NNC, Cheung HM, et al. Selective dorsal rhizotomy in Hong Kong: Multidimensional outcome measures. Pediatric Neurology. 2008;39(1):22-32.

22. Chen J, Wang Y, Liu Y, Yang Y, Ma Y, Wang S. [Individualized neurosurgical treatments of spastic cerebral palsy]. Zhonghua yi xue za zhi. 2014;94(5):376-8.

23. Chen S, Xie Z, Xu J, Li H, Zhang M, Ding C, et al. Analysis of complications of selective posterior rhizotomy combined with replacement laminoplasty for the treatment of spastic cerebral palsy. Chinese Journal of Neurosurgery. 2023;39(1):55

24. Chicoine MR, Park TS, Kaufman BA. Selective dorsal rhizotomy and rates of orthopedic surgery in children with spastic cerebral palsy. Journal of Neurosurgery. 1997;86(1):34-9.

25. Chicoine MR, Park TS, Vogler GP, Kaufman BA. Predictors of ability to walk after selective dorsal rhizotomy in children with cerebral palsy. Neurosurgery. 1996;38(4):711-4.

26. Chiu PKF, Yam KY, Lam TY, Cheng CH, Yu C, Li ML, et al. Does selective dorsal rhizotomy improve bladder function in children with cerebral palsy? International Urology and Nephrology. 2014;46(10):1929-33.

27. Chow CP, Wong LY, Poon CYC, Yiu BPH, Wong TPS, Wong M, et al. Functional outcome after selective dorsal rhizotomy: a retrospective case control study. Childs Nervous System. 2024;40(3):625-34.

28. Chugh D, Cawker SP, Katchburian L, Carr LJ, Aquilina K, Morgan S, et al. Quality of Movement Changes in Ambulatory Children with Cerebral Palsy 1 Year after Selective Dorsal Rhizotomy. Physiotherapy Canada. 2024.

29. Chugh D, Waite G, Harniess P, Oulton K, Wray J, Cawker S. 'I Didn't Know What Was Going to Happen': Children's and Young People's Experiences and Their Involvement Before and After Selective Dorsal Rhizotomy. Physical & Occupational Therapy in Pediatrics. 2024.

30. Cobb MA, Boop FA. Replacement laminoplasty in selective dorsal rhizotomy: possible protection against the development of musculoskeletal pain. Pediatric neurosurgery. 1994;21(4):237-42.

31. Cole GF, Farmer SE, Roberts A, Stewart C, Patrick JH. Selective dorsal rhizotomy for children with cerebral palsy: the Oswestry experience. Archives of Disease in Childhood. 2007;92(9):781-5.

32. Craft S, Park TS, White DA, Schatz J, Noetzel M, Arnold S. Changes in cognitive performance in children with spastic diplegic cerebral palsy following selective dorsal rhizotomy. Pediatric Neurosurgery. 1995;23(2):68-75.

33. Daunter AK, Kratz AL, Hurvitz EA. Long-term impact of childhood selective dorsal rhizotomy on pain, fatigue, and function: a case–control study. Developmental Medicine and Child Neurology. 2017;59(10):1089-95.

34. de Monaco BA, Candido AAD, Teixeira MJ, Alho EJL. Impact of selective dorsal rhizotomy to cerebral palsy children caregivers' burden. Childs Nervous System. 2024;40(5):1461-9.

35. Dekopov AV, Tomsky AA, Gaevyi IO, Salova EM, Kozlova AB, Ogurtsova AA, et al. Long-term outcomes of SDR in various groups of cerebral palsy (CP) patients. Zhurnal voprosy neirokhirurgii imeni N N Burdenko. 2015;79(6):29

36. Duc Lien N, Van Linh N, Cam Van NT, Giang LT, King DT, Tarren A, et al. Selective Dorsal Rhizotomy for Spastic Cerebral Palsy: Report of 18 Cases Performed in the North of Vietnam. World Neurosurgery. 2024;188:e128-e33.

37. Dudgeon BJ, Libby AK, McLaughlin JF, Hays RM, Bjornson KF, Roberts TS. Prospective measurement of functional changes after selective dorsal rhizotomy. Archives of Physical Medicine and Rehabilitation. 1994;75(1):46-53.

38. Dudley RWR, Parolin M, Gagnon B, Saluja R, Yap R, Montpetit K, et al. Long-term functional benefits of selective dorsal rhizotomy for spastic cerebral palsy Clinical article. Journal of Neurosurgery-Pediatrics. 2013;12(2):142-50.

39. Duffy EA, Hornung AL, Chen BP-J, Munger ME, Aldahondo N, Krach LE, et al. Comparing short-term outcomes between conus medullaris and cauda equina surgical techniques of selective dorsal rhizotomy. Developmental medicine and child neurology. 2021;63(3):336-42.

40. Eliasson AC, Öhrvall AM, Borell L. Parents' perspectives of changes in movement affecting daily life following selective dorsal rhizotomy in children with cerebral palsy. Physical and Occupational Therapy in Pediatrics. 2000;19(3-4):91-109.

41. Engsberg JR, Ross SA, Collins DR, Park TS. Predicting functional change from preintervention measures in selective dorsal rhizotomy. Journal of Neurosurgery. 2007;106(4):282-7.

42. Engsberg JR, Ross SA, Collins DR, Tae SP. Effect of selective dorsal rhizotomy in the treatment of children with cerebral palsy. Journal of Neurosurgery. 2006;105 PEDIATRICS(SUPPL. 1):8-15.

43. Engsberg LR, Ross SA, Park TS. Quantifying active ankle range of motion in cerebral palsy following selective dorsal rhizotomy. Journal of Applied Biomechanics. 2004;20(1):103-11.

44. Engsberg JR, Ross SA, Wagner JM, Park TS, Engsberg JR, Ross SA, et al. Changes in hip spasticity and strength following selective dorsal rhizotomy and physical therapy for spastic cerebral palsy. Developmental Medicine & Child Neurology. 2002;44(4):220-6.

45. Engsberg JR, Ross SA, Park TS. Changes in ankle spasticity and strength following selective dorsal rhizotomy and physical therapy for spastic cerebral palsy. Journal of Neurosurgery. 1999;91(5):727-32.

46. Engsberg JR, Olree KS, Ross SA, Park TS. Spasticity and strength changes as a function of selective dorsal rhizotomy. Journal of Neurosurgery. 1998;88(6):1020-6.

47. Feger MA, Lunsford CD, Sauer LD, Novicoff W, Abel MF. Comparative Effects of Multilevel Muscle Tendon Surgery, Osteotomies, and Dorsal Rhizotomy on Functional and Gait Outcome Measures for Children With Cerebral Palsy. Pm&R. 2015;7(5):485-93.

48. Floeter N, Lebek S, Bakir MS, Sarpong A, Wagner C, Haberl EJ, et al. Changes in hip geometry after selective dorsal rhizotomy in children with cerebral palsy. Hip International. 2014;24(6):638-43.

49. Forst H, Sylvanus T. Impact of Occupational Therapy on Self-Care After Selective Dorsal Rhizotomy Among Children With Cerebral Palsy. American Journal of Occupational Therapy. 2022;76(6).

50. Funk JF, Haberl H. Monosegmental laminoplasty for selective dorsal rhizotomy--operative technique and influence on the development of scoliosis in ambulatory children with cerebral palsy. Child's nervous system : ChNS : official journal of the International Society for Pediatric Neurosurgery. 2016;32(5):819-25.

51. Funk JF, Panthen A, Bakir MS, Gruschke F, Sarpong A, Wagner C, et al. Predictors for the benefit of selective dorsal rhizotomy. Research in Developmental Disabilities. 2015;37:127-34.

52. Gadgil N, Schwabe AL, Wright E, Barbuto A, Dugan EL, Thomas SP, et al. Focal selective dorsal rhizotomy and concurrent deformity correction: a combined approach. Journal of neurosurgery Pediatrics. 2024:1-8.

53. Galarza M, Fowler EG, Chipps L, Padden TM, Lazareff JA. Functional assessment of children with cerebral palsy following limited (L4-S1) selective posterior rhizotomy--a preliminary report. Acta neurochirurgica. 2001;143(9):865-72.

54. Gigante P, McDowell MM, Bruce SS, Chirelstein G, Cihriboga CA, Dutkowsky J, et al. Reduction in upper-extremity tone after lumbar selective dorsal rhizotomy in children with spastic cerebral palsy. Journal of Neurosurgery-Pediatrics. 2013;12(6):588-94.

55. Gillespie CS, George AM, Hall B, Toh S, Islim AI, Hennigan D, et al. The effect of GMFCS level, age, sex, and dystonia on multi-dimensional outcomes after selective dorsal rhizotomy: prospective observational study. Childs Nervous System. 2021;37(5):1729-40.

56. Gimarc K, Yandow S, Browd S, Leibow C, Pham K. Combined Selective Dorsal Rhizotomy and Single-Event Multilevel Surgery in a Child with Spastic Diplegic Cerebral Palsy: A Case Report. Pediatric Neurosurgery. 2021.

57. Golan JD, Hall JA, O'Gorman G, Poulin C, Benaroch TE, Cantin M-A, et al. Spinal deformities following selective dorsal rhizotomy. Journal of neurosurgery. 2007;106(6 Suppl):441-9.

58. Gooch JL, Walker ML. Spinal stenosis after total lumbar laminectomy for selective dorsal rhizotomy. Pediatric Neurosurgery. 1996;25(1):28-30.

59. Graubert C, Song KM, McLaughlin JF, Bjornson KF. Changes in gait at 1 year post-selective dorsal rhizotomy: Results of a prospective randomized study. Journal of Pediatric Orthopaedics. 2000;20(4):496-500.

60. Grootveld LR, van Schie PEM, Buizer AI, Jeroen Vermeulen R, van Ouwerkerk WJR, Strijers RLM, et al. Sudden falls as a persistent complication of selective dorsal rhizotomy surgery in children with bilateral spasticity: report of 3 cases. Journal of neurosurgery Pediatrics. 2016;18(2):192-5.

61. Grunt S, Henneman WJP, Bakker MJ, Harlaar J, Van Der Ouwerkerk WJR, Van Schie P, et al. Effect of selective dorsal rhizotomy on gait in children with bilateral spastic paresis: Kinematic and EMG-pattern changes. Neuropediatrics. 2010;41(5):209-16.

62. Grunt S, Becher JG, van Schie P, van Ouwerkerk WJR, Ahmadi M, Vermeulen RJ. Preoperative MRI findings and functional outcome after selective dorsal rhizotomy in children with bilateral spasticity. Childs Nervous System. 2010;26(2):191-8.

63. Gul SM, Steinbok P, McLeod K. Long-term outcome after selective posterior rhizotomy in children with spastic cerebral palsy. Pediatric Neurosurgery. 1999;31(2):84-95.

64. Gutknecht SM, Schwartz MH, Munger ME. Ambulatory children with cerebral palsy do not exhibit unhealthy weight gain following selective dorsal rhizotomy. Developmental Medicine and Child Neurology. 2015;57(11):1070-5.

65. Hays RM, McLaughlin JF, Bjornson KF, Stephens K, Roberts TS, Price R, et al. Electrophysiological monitoring during selective dorsal rhizotomy, and spasticity and GMFM performance. Developmental Medicine & Child Neurology. 1998;40(4):233-8.

66. He W, Xu M, Wang MC, Han SP, Yang J. Management of lower limbs spasm following cerebral palsy using selective posterior rhizotomy. Chinese Journal of Clinical Rehabilitation. 2002;6(6):914-5.

67. He ZX, Wong ST, Law HY, Lao LMM, Chan KFH, Chan NCN, et al. Multidimensional Outcomes of Selective Dorsal Rhizotomy for Children With Spastic Cerebral Palsy: Single-Level Laminectomy vs Multiple-Level Laminotomy Techniques. Neurosurgery. 2022;91(3):513-24.

68. Heim RC, Park TS, Vogler GP, Kaufman BA, Noetzel MJ, Ortman MR. Changes in hip migration after selective dorsal rhizotomy for spastic quadriplegia in cerebral palsy. Journal of Neurosurgery. 1995;82(4):567-71.

69. Hicdonmez T, Steinbok P, Beauchamp R, Sawatzky B. Hip joint subluxation after selective dorsal rhizotomy for spastic cerebral palsy. Journal of Neurosurgery. 2005;103(1):10-6.

70. Hodgkinson I, Berard C, Jindrich ML, Sindou M, Mertens P, Berard J. Selective dorsal rhizotomy in children with cerebral palsy. Results in 18 cases at one year postoperatively. Stereotactic and functional neurosurgery. 1997;69(1-4 Pt 2):259-67.

71. Horínek D, Hoza D, Cerny R, Vyhnálek M, Sturm D, Bojar M, et al. Two cases of improvement of smooth pursuit eye movements after selective posterior rhizotomy. Childs Nervous System. 2008;24(11):1283-8.

72. Houle AM, Vernet O, Jednak R, Salle JLP, Farmer JP. Bladder function before and after selective dorsal rhizotomy in children with cerebral palsy. Journal of Urology. 1998;160(3):1088-91.

73. Hoza D, Lastovka M, Bojar M, Cerny R, Sturm D, Kraus J, et al. An Improvement in Smooth Pursuit Eye Movements and Phonation Following Selective Dorsal Rhizotomy. Ceska a Slovenska Neurologie a Neurochirurgie. 2009;72(4):378-82.

74. Hurvitz EA, Marciniak CM, Daunter AK, Haapala HJ, Stibb SM, McCormick SF, et al. Functional outcomes of childhood dorsal rhizotomy in adults and adolescents with cerebral palsy Clinical article. Journal of Neurosurgery-Pediatrics. 2013;11(4):380-8.

75. Illum NO, Torp-Pedersen L, Midholm S, Selmar PE, Simesen K. Selective dorsal rhizotomy for children with severe spastic cerebral diplegia. Ugeskrift for Laeger. 2006;168(8):785-9.

76. Iorio-Morin C, Yap R, Dudley RWR, Poulin C, Cantin M-A, Benaroch TE, et al. Selective Dorsal Root Rhizotomy for Spastic Cerebral Palsy: A Longitudinal Case-Control Analysis of Functional Outcome. Neurosurgery. 2020;87(2):186-92.

77. Jeffery SMT, Markia B, Pople IK, Aquilina K, Smith J, Mohamed AZ, et al. Surgical Outcomes of Single-Level Bilateral Selective Dorsal Rhizotomy for Spastic Diplegia in 150 Consecutive Patients. World Neurosurgery. 2019;125:E60-E6.

78. Jia Y, Xiao Y, Yang W. [Analysis of medium- and long-term effectiveness of selective posterior rhizotomy for spastic cerebral palsy]. Zhongguo xiu fu chong jian wai ke za zhi = Zhongguo xiufu chongjian waike zazhi = Chinese journal of reparative and reconstructive surgery. 2013;27(11):1345

79. Jiang WB, Zhang L, Wei M, Wang R, Xiao B, Wang JL, et al. A preliminary study on the spasticity reduction of quadriceps after selective dorsal rhizotomy in pediatric cases of spastic cerebral palsy. Acta Neurochirurgica. 2024;166(1).

80. Jiang W, Jiang S, Yu Y, Zhan Q, Wei M, Mei R, et al. Improvement of the gait pattern after selective dorsal rhizotomy derives from changes of kinematic parameters in the sagittal plane. Frontiers in Pediatrics. 2022;10.

81. Johnson MB, Goldstein L, Thomas SS, Piatt J, Aiona M, Sussman M. Spinal deformity after selective dorsal rhizotomy in ambulatory patients with cerebral palsy. Journal of Pediatric Orthopaedics. 2004;24(5):529-36.

82. Josenby AL, Wagner P, Jarnlo G-B, Westbom L, Nordmark E. Functional performance in self-care and mobility after selective dorsal rhizotomy: a 10-year practice-based follow-up study. Developmental medicine and child neurology. 2015;57(3):286-93.

83. Josenby AL, Wagner P, Jarnlo G-B, Westbom L, Nordmark E. Motor function after selective dorsal rhizotomy: a 10-year practice-based follow-up study. Developmental medicine and child neurology. 2012;54(5):429-35.

84. Kainz H, Hoang H, Pitto L, Wesseling M, Van Rossom S, Van Campenhout A, et al. Selective dorsal rhizotomy improves muscle forces during walking in children with spastic cerebral palsy. Clinical Biomechanics. 2019;65:26-33.

85. Kan P, Gooch J, Amini A, Ploeger D, Grams B, Oberg W, et al. Surgical treatment of spasticity in children: comparison of selective dorsal rhizotomy and intrathecal baclofen pump implantation. Childs Nervous System. 2008;24(2):239-43.

86. Kaufman HH, Bodensteiner J, Burkart B, Gutmann L, Kopitnik T, Hochberg V, et al. Treatment of spastic gait in cerebral palsy. The West Virginia medical journal. 1994;90(5):190-2.

87. Kenis VM, Ivanov SV, Kiseleva TI. Selective dorsal rhizotomy opportunities with foot deformities in children with cerebral palsy. Pediatric Traumatology, Orthopaedics and Reconstructive Surgery. 2015;3(1):22-6.

88. Kim DS, Choi JU, Yang KH, Park CI. Selective posterior rhizotomy in children with cerebral palsy: a 10-year experience. Childs Nervous System. 2001;17(9):556-62.

89. Kim DS, Choi JU, Yang KH, Park CI, Park ES. Selective posterior rhizotomy for lower extremity spasticity: How much and which of the posterior rootlets should be cut? Surgical Neurology. 2002;57(2):87-93.

90. Kim HS, Steinbok P, Wickenheiser D. Predictors of poor outcome after selective dorsal rhizotomy in treatment of spastic cerebral palsy. Childs Nervous System. 2006;22(1):60-6.

91. Langerak NG, Veerbeek BE, Fieggen AG, Lamberts RP. Gait status 26–35 years after selective dorsal rhizotomy: A 9 year follow up study. Gait and Posture. 2022;91:284-9.

92. Langerak NG, Tam N, Vaughan CL, Fieggen AG, Schwartz MH. Gait status 17-26 years after selective dorsal rhizotomy. Gait & Posture. 2012;35(2):244-9.

93. Langerak NG, Hillier SL, Verkoeijen PP, Peter JC, Fieggen AG, Vaughan CL. LEVEL OF ACTIVITY AND PARTICIPATION IN ADULTS WITH SPASTIC DIPLEGIA 17-26 YEARS AFTER SELECTIVE DORSAL RHIZOTOMY. Journal of Rehabilitation Medicine. 2011;43(4):330-7.

94. Langerak NG, Vaughan CL, Hoffman EB, Figaji AA, Fieggen AG, Peter JC. Incidence of spinal abnormalities in patients with spastic diplegia 17 to 26 years after selective dorsal rhizotomy. Childs Nervous System. 2009;25(12):1593-603.

95. Langerak NG, Lamberts RP, Fieggen AG, Peter JC, Peacock WJ, Vaughan CL. Functional Status of Patients With Cerebral Palsy According to the International Classification of Functioning, Disability and Health Model: A 20-Year Follow-Up Study After Selective Dorsal Rhizotomy. Archives of Physical Medicine and Rehabilitation. 2009;90(6):994-1003.

96. Langerak NG, Lamberts RP, Fieggen AG, Peter JC, van der Merwe L, Peacock WJ, et al. A prospective gait analysis study in patients with diplegic cerebral palsy 20 years after selective dorsal rhizotomy. Journal of Neurosurgery-Pediatrics. 2008;1(3):180-6.

97. Lazareff JA, Garcia-Mendez MA, De Rosa R, Olmstead C. Limited (L4-S1, L5-S1) selective dorsal rhizotomy for reducing spasticity in cerebral palsy. Acta Neurochirurgica. 1999;141(7):743-52.

98. Leland Albright A, Barry MJ, Fasick MP, Janosky J. Effects of continuous intrathecal baclofen infusion and selective posterior rhizotomy on upper extremity spasticity. Pediatric Neurosurgery. 1995;23(2):82-5.

99. Lewin JE, Mix CM, Gaebler-Spira D. Self-help and upper extremity changes in 36 children with cerebral palsy subsequent to selective posterior rhizotomy and intensive occupational and physical therapy. Physical and Occupational Therapy in Pediatrics. 1994;13(3):25-42.

100. Lewis JA, Bear N, Smith N, Baker F, Lee OS, Wynter M, et al. Goal setting, goal attainment and quality of life of children following selective dorsal rhizotomy. Child: Care, Health and Development. 2023;49(4):760-8.

101. Li Z, Zhu J, Liu X. Deformity of lumbar spine after selective dorsal rhizotomy for spastic cerebral palsy. Microsurgery. 2008;28(1):10-2.

102. Lian YY, Jin YH, Li WQ, Zhang J, Pei FX. Treatment of spastic cerebral palsy by selective posterior rhizotomy combined with orthopaedic operation of lower extremities. Chinese Journal of Clinical Rehabilitation. 2004;8(33):7433

103. Limpaphayom N, Stewart S, Wang L, Liu J, Park TS, Dobbs MB. Functional outcomes after selective dorsal rhizotomy followed by minimally invasive tendon lengthening procedures in children with spastic cerebral palsy. Journal of Pediatric Orthopaedics-Part B. 2020;29(1):1-8.

104. Loewen P, Steinbok P, Holsti L, MacKay M. Upper extremity performance and self-care skill changes in children with spastic cerebral palsy following selective posterior rhizotomy. Pediatric Neurosurgery. 1998;29(4):191-8.

105. Lundkvist Josenby A, Westbom L. No support that early selective dorsal rhizotomy increase frequency of scoliosis and spinal pain - a longitudinal population-based register study from four to 25 years of age. BMC Musculoskeletal Disorders. 2020;21(1):N.PAG-N.PAG.

106. Lundkvist A, Hägglund G. Orthopaedic surgery after selective dorsal rhizotomy. Journal of Pediatric Orthopaedics-Part B. 2006;15(4):244-6.

107. MacWilliams BA, McMulkin ML, Duffy EA, Munger ME, Chen BPJ, Novacheck TF, et al. Long-term effects of spasticity treatment, including selective dorsal rhizotomy, for individuals with cerebral palsy. Developmental Medicine and Child Neurology. 2022;64(5):561-8.

108. Mäenpää H, Salokorpi T, Jaakkola R, Blomstedt G, Sainio K, Merikanto J, et al. Follow-up of children with cerebral palsy after selective posterior rhizotomy with intensive physiotherapy or physiotherapy alone. Neuropediatrics. 2003;34(2):67-71.

109. Mantese B, Pirozzi Chiusa CG, Basilotta Marquez Y, Gotter Campo MP, Nazar R, Crespo M, et al. Selective dorsal rhizotomy: Analysis of two rootlet sectioning techniques. Child's Nervous System. 2024;40(4):1147-57.

110. Marron A, O'Sullivan R, Leonard J, Kiernan D. The medium-term effects of selective dorsal rhizotomy on gait compared to a matched cerebral palsy non-SDR group: A follow-up study. Gait & Posture. 2023;99:124-32.

111. Marty GR, Dias LS, Gaebler-Spira D. Selective posterior rhizotomy and soft-tissue procedures for the treatment of cerebral diplegia. Journal of Bone and Joint Surgery. 1995;77(5):713-8.

112. McFall J, Stewart C, Kidgell V, Postans N, Jarvis S, Freeman R, et al. Changes in gait which occur before and during the adolescent growth spurt in children treated by selective dorsal rhizotomy. Gait & Posture. 2015;42(3):317-22.

113. McLaughlin JF, Felix SD, Nowbar S, Ferrel A, Bjornson K, Hays RM. Lower extremity sensory function in children with cerebral palsy. Pediatric Rehabilitation. 2005;8(1):45-52.

114. McLaughlin JF, Bjornson KF, Astley SJ, Graubert C, Hays RM, Roberts TS, et al. Selective dorsal rhizotomy: efficacy and safety in an investigator-masked randomized clinical trial. Developmental Medicine & Child Neurology. 1998;40(4):220-32.

115. McLaughlin JF, Bjornson KF, Astley SJ, Hays RM, Hoffinger SA, Armantrout EA, et al. The role of selective dorsal rhizotomy in cerebral palsy: critical evaluation of a prospective clinical series. Developmental medicine and child neurology. 1994;36(9):755‐69.

116. McMulkin ML, MacWilliams BA, Nelson EA, Munger ME, Chen BP-J, Novacheck TF, et al. The long-term effects of aggressive spasticity reducing treatment, including selective dorsal rhizotomy, on joint kinematic outcomes of persons with cerebral palsy. Gait & posture. 2023;105:139-48.

117. Macwilliams BA, Johnson BA, Shuckra AL, D'Astous JL. Functional decline in children undergoing selective dorsal rhizotomy after age 10. Developmental Medicine and Child Neurology. 2011;53(8):717-23.

118. Miller SD, Juricic M, Bone JN, Steinbok P, Mulpuri K. The Effect of Selective Dorsal Rhizotomy on Hip Displacement in Children With Cerebral Palsy A Long-term Follow-up Study. Journal of Pediatric Orthopaedics. 2023;43(9):E701-E6.

119. Miller AC, JohannMurphy M, PittenCate IM. Pain, anxiety, and cooperativeness in children with cerebral palsy after rhizotomy: Changes throughout rehabilitation. Journal of Pediatric Psychology. 1997;22(5):689-705.

120. Mittal S, Farmer JP, Al-Atassi B, Gibis J, Kennedy E, Galli C, et al. Long-term functional outcome after selective posterior rhizotomy. Journal of Neurosurgery. 2002;97(2):315-25.

121. Mittal S, Farmer JP, Al-Atassi B, Montpetit K, Gervais N, Poulin C, et al. Functional performance following selective posterior rhizotomy: long-term results determined using a validated evaluative measure. Journal of Neurosurgery. 2002;97(3):510-8.

122. Mittal S, Farmer JP, Al-Atassi B, Montpetit K, Gervais N, Poulin C, et al. Impact of selective posterior rhizotomy on fine motor skills: Long-term results using a validated evaluative measure. Pediatric Neurosurgery. 2002;36(3):133-41.

123. Morota N. Functional posterior rhizotomy: the Tokyo experience. Childs Nervous System. 2007;23(9):1007-14.

124. Morota N, Abbott R, Kofler M, Epstein FJ, Cohen H. RESIDUAL SPASTICITY AFTER SELECTIVE POSTERIOR RHIZOTOMY. Childs Nervous System. 1995;11(3):161-5.

125. Mortenson P, Sadashiva N, Tamber MS, Steinbok P. Long-term upper extremity performance in children with cerebral palsy following selective dorsal rhizotomy. Childs Nervous System. 2021;37(6):1983-9.

126. Mu XH, Xu L, Xu SG, Cao X, Zhang P, Zheng CY, et al. Application of exercise therapy on rehabilitation after selective posterior rhizotomy (SPR) in children with cerebral palsy. Zhongguo gu shang = China journal of orthopaedics and traumatology. 2009;22(9):674

127. Munger ME, Aldahondo N, Krach LE, Novacheck TF, Schwartz MH. Long-term outcomes after selective dorsal rhizotomy: a retrospective matched cohort study. Developmental Medicine and Child Neurology. 2017;59(11):1196-203.

128. Niedzwecki C, Barbuto A, Mitchell K, Wirt S, Seymour M, Thomas S, et al. Comparison of outcomes following surgical intervention and inpatient rehabilitation stays in children with cerebral palsy. Pm&R. 2024;16(5):449-61.

129. Nishida T, Thatcher SW, Marty GR. Selective posterior rhizotomy for children with cerebral palsy: a 7-year experience. Child's Nervous System. 1995;11(7):374-80.

130. Nordmark E, Josenby AL, Lagergren J, Andersson G, Strömblad LG, Westbom L, et al. Long-term outcomes five years after selective dorsal rhizotomy. BMC Pediatrics. 2008;8:54-.

131. Nordmark E, Jarnlo GB, Hägglund G. Comparison of the gross motor function measure and paediatric evaluation of disability inventory in assessing motor function in children undergoing selective dorsal rhizotomy. Developmental Medicine and Child Neurology. 2000;42(4):245-52.

132. O'Brien DF, Tae SP, Puglisi JA, Collins DR, Leuthardt EC, Leonard JR. Orthopedic surgery after selective dorsal rhizotomy for spastic diplegia in relation to ambulatory status and age. Journal of Neurosurgery. 2005;103 PEDIATRICS(SUPPL. 1):5-9.

133. O'Brien DF, Park TS, Puglisi JA, Collins DR, Leuthardt EC. Effect of selective dorsal rhizotomy on need for orthopedic surgery for spastic quadriplegic cerebral palsy: long-term outcome analysis in relation to age. Journal of Neurosurgery. 2004;101(1):59-63.

134. O’Sullivan R, Leonard J, Quinn A, Kiernan D. The short-term effects of selective dorsal rhizotomy on gait compared to matched cerebral palsy control groups. PLoS ONE. 2019;14(7).

135. Olree KS, Engsberg JR, Ross SA, Park TS, Olree KS, Engsberg JR, et al. Changes in synergistic movement patterns after selective dorsal rhizotomy. Developmental Medicine & Child Neurology. 2000;42(5):297-303.

136. Ou C, Kent S, Miller S, Steinbok P. Selective dorsal rhizotomy in children: comparison of outcomes after single-level versus multi-level laminectomy technique. Canadian journal of neuroscience nursing. 2010;32(3):17-24.

137. Oudenhoven LM, van der Krogt MM, Romei M, van Schie PEM, van de Pol LA, van Ouwerkerk WJR, et al. Factors Associated With Long-Term Improvement of Gait After Selective Dorsal Rhizotomy. Archives of Physical Medicine and Rehabilitation. 2019;100(3):474-80.

138. Park TS, Joh S, Walter DM, Meyer NL. Parent-Reported Outcomes of Early Childhood Selective Dorsal Rhizotomy for the Treatment of Spastic Diplegia. Cureus Journal of Medical Science. 2021;13(6).

139. Park TS, Joh S, Walter DM, Meyer NL, Dobbs MB. Selective Dorsal Rhizotomy for the Treatment of Spastic Hemiplegic Cerebral Palsy. Cureus Journal of Medical Science. 2020;12(8).

140. Park TS, Joh S, Walter DM, Dobbs MB. Selective Dorsal Rhizotomy for the Treatment of Spastic Triplegic Cerebral Palsy. Cureus Journal of Medical Science. 2020;12(7).

141. Park TS, Miller BA, Cho J. Simultaneous Selective Dorsal Rhizotomy and Baclofen Pump Removal Improve Ambulation in Patients with Spastic Cerebral Palsy. Cureus Journal of Medical Science. 2018;10(6).

142. Park TS, Liu JL, Edwards C, Walter DM, Dobbs MB. Functional Outcomes of Childhood Selective Dorsal Rhizotomy 20 to 28 Years Later. Cureus Journal of Medical Science. 2017;9(5).

143. Park TS, Edwards C, Liu EL, Walter DM, Dobbs MB. Beneficial Effects of Childhood Selective Dorsal Rhizotomy in Adulthood. Cureus Journal of Medical Science. 2017;9(3).

144. Park TS, Vogler GP, Phillips LH, Kaufman BA, Ortman MR, McClure SM, et al. Effects of selective dorsal rhizotomy for spastic diplegia on hip migration in cerebral palsy. Pediatric Neurosurgery. 1994;20(1):43-9.

145. Peter JC, Hoffman EB, Arens LJ. Spondylolysis and spondylolisthesis after five-level lumbosacral laminectomy for selective posterior rhizotomy in cerebral palsy. Child's Nervous System. 1993;9(5):285-8.

146. Peter JC, Arens LJ. Selective posterior lumbosacral rhizotomy in teenagers and Young adults with spastic cerebral palsy. British Journal of Neurosurgery. 1994;8(2):135-9.

147. Peter JC, Arens LJ. Selective posterior lumbosacral rhizotomy for the management of cerebral palsy spasticity. A 10-year experience. South African Medical Journal. 1993;83(10):745-7.

148. Qijia Z, Xidan Y, Bo X, Min S, Wenbin J, Min W, et al. Efficacy of novel protocol guided single-level laminectomy selective dorsal rhizotomy plus intensive rehabilitation for children with spastic cerebral palsy. Chinese Journal of Pediatric Surgery. 2019;40(10):886-92.

149. Qijia Z, Shuihua W, Bo X, Wenbing J, Min W, Junin W, et al. Short-term efficacy of single-level laminectomy selective dorsal rhizotomy guided by novel neuroelectro- physiological monitoring protocol for children with spastic cerebral palsy: a multicenter retrospective clinical study. Journal of Clinical Pediatric Surgery. 2022;21(6):523

150. Ravera EP, Rozumalski A. Selective dorsal rhizotomy and its effect on muscle force during walking: A comprehensive study. Journal of Biomechanics. 2024;164.

151. Ravindra VM, Christensen MT, Onwuzulike K, Smith JT, Halvorson K, Brockmeyer DL, et al. Risk factors for progressive neuromuscular scoliosis requiring posterior spinal fusion after selective dorsal rhizotomy. Journal of Neurosurgery-Pediatrics. 2017;20(5):456-63.

152. Robins JMW, Boyle A, McCune K, Lodh R, Goodden JR. Quality of life after selective dorsal rhizotomy: an assessment of family-reported outcomes using the CPQoL questionnaire. Childs Nervous System. 2020;36(9):1977-83.

153. Romei M, Oudenhoven LM, van Schie PEM, van Ouwerkerk WJR, van der Krogt MM, Buizer AI. Evolution of gait in adolescents and young adults with spastic diplegia after selective dorsal rhizotomy in childhood: A 10 year follow-up study. Gait & Posture. 2018;64:108-13.

154. Ross SA, Engsberg JR, Olree KS, Park TS. Quadriceps and hamstring strength changes as a function of selective dorsal rhizotomy surgery and rehabilitation. Pediatric Physical Therapy. 2001;13(1):2-9.

155. Rumberg F, Bakir MS, Taylor WR, Haberl H, Sarpong A, Sharankou I, et al. The Effects of Selective Dorsal Rhizotomy on Balance and Symmetry of Gait in Children with Cerebral Palsy (vol 4, e0152930, 2016). Plos One. 2017;12(5).

156. Sacco DJ, Tylkowski CM, Warf BC. Nonselective partial dorsal rhizotomy: A clinical experience with 1-year follow-up. Pediatric Neurosurgery. 2000;32(3):114-8.

157. Sagan L, Ogłodziński J, Madany K, Jarosz M, Lickendorf M, Małasiak P, et al. ffect of selective dorsal rhizotomy for treatment of spasticity related to cerebral palsy - report on one-year postoperative results in the Polish population. Child Neurology. 2020;56(29):19-26.

158. Santos MV, Carneiro VM, Oliveira PNBGC, Caldas CAT, MacHado HR. Surgical results of selective dorsal rhizotomy for the treatment of spastic cerebral palsy. Journal of Pediatric Neurosciences. 2021;16(1):24-9.

159. Sargut TA, Haberl H, Wolter S, Tafelski S, van Riesen A, Linhard M, et al. Motor and functional outcome of selective dorsal rhizotomy in children with spastic diplegia at 12 and 24 months of follow-up. Acta neurochirurgica. 2021;163(10):2837-44.

160. Schwartz MH, Viehweger E, Stout J, Novacheck TF, Gage JR. Comprehensive Treatment of Ambulatory Children with Cerebral Palsy: An Outcome Assessment. Journal of Pediatric Orthopaedics. 2004;24(1):45-53.

161. Shevelev IN, Shabalov VA, Artarian AA, Safronov VA, Stepanenko AI. The use of selective dorsal rhizotomy for treating spasticity in patients with infantile cerebral palsy. Zhurnal voprosy neirokhirurgii imeni N N Burdenko. 1996(3):19-22.

162. Shuman BR, Goudriaan M, Desloovere K, Schwartz MH, Steele KM. Muscle synergies demonstrate only minimal changes after treatment in cerebral palsy. Journal of Neuroengineering and Rehabilitation. 2019;16.

163. Spazzapan P, Bosnjak R, Rodi Z, Kos N, Groleger K, Velnar T. Selective dorsal rhizotomy: short-term results and early experiences with a newly established surgical treatment in Slovenia. Journal of Integrative Neuroscience. 2022;21(3).

164. Spiegel DA, Loder RT, Alley KA, Rowley S, Gutknecht S, Smith-Wright DL, et al. Spinal Deformity Following Selective Dorsal Rhizotomy. Journal of Pediatric Orthopaedics. 2004;24(1):30-6.

165. Spijker M, Strijers RLM, van Ouwerkerk WJR, Becher JG. Disappearance of Spasticity After Selective Dorsal Rhizotomy Does Not Prevent Muscle Shortening in Children With Cerebral Palsy: A Case Report. Journal of Child Neurology. 2009;24(5):625-7.

166. Staudt LA, Nuwer MR, Peacock WJ. Intraoperative monitoring during selective posterior rhizotomy: Technique and patient outcome. Electromyography and Motor Control-Electroencephalography and Clinical Neurophysiology. 1995;97(6):296-309.

167. Steinbok P, Tidemann AJ, Miller S, Mortenson P, Bowen-Roberts T. Electrophysiologically guided versus non-electrophysiologically guided selective dorsal rhizotomy for spastic cerebral palsy: a comparison of outcomes. Childs Nervous System. 2009;25(9):1091-6.

168. Steinbok P, Hicdonmez T, Sawatzky B, Beauchamp R, Wickenheiser D. Spinal deformities after selective dorsal rhizotomy for spastic cerebral palsy. Journal of Neurosurgery. 2005;102(4):363-73.

169. Steinbok P, Reiner A, Kestle JR. Therapeutic electrical stimulation following selective posterior rhizotomy in children with spastic diplegic cerebral palsy: a randomized clinical trial. Developmental medicine and child neurology. 1997;39(8):515‐20.

170. Steinbok P, Reiner AM, Beauchamp R, Armstrong RW, Cochrane DD, Kestle J. A randomized clinical trial to compare selective posterior rhizotomy plus physiotherapy with physiotherapy alone in children with spastic diplegic cerebral palsy. Developmental medicine and child neurology. 1997;39(3):178‐84.

171. Steinbok P, Gustavsson B, Kestle JR, Reiner A, Cochrane DD. Relationship of intraoperative electrophysiological criteria to outcome after selective functional posterior rhizotomy. Journal of neurosurgery. 1995;83(1):18-26.

172. Steinbok P, McLeod K. Comparison of motor outcomes after selective dorsal rhizotomy with and without preoperative intensified physiotherapy in children with spastic diplegic cerebral palsy. Pediatric Neurosurgery. 2002;36(3):142-7.

173. Steinbok P, Schrag C. Complications after selective posterior rhizotomy for spasticity in children with cerebral palsy. Pediatric Neurosurgery. 1998;28(6):300-13.

174. Stepanenko AI, Shabalov VA, Shevelev IN, Arkhipova NA, Artarian AA, Blinkov SM, et al. The effect of selective dorsal rhizotomy on motor function in patients with infantile cerebral palsy. Zhurnal Voprosy Nejrokhirurgii Imeni NN Burdenko. 1999(4):14-7;discussion7.

175. Subramanian N, Vaughan CL, Peter JC, Arens LJ. Gait before and 10 years after rhizotomy in children with cerebral palsy spasticity. Journal of Neurosurgery. 1998;88(6):1014-9.

176. Summers J, Coker B, Eddy S, Elstad M, Bunce C, Bourmpaki E, et al. Selective dorsal rhizotomy in ambulant children with cerebral palsy: an observational cohort study. Lancet Child & Adolescent Health. 2019;3(7):455-62.

177. Sweetser PM, Badell A, Schneider S, Badlani GH. Effects of sacral dorsal rhizotomy on bladder function in patients with spastic cerebral palsy. Neurourology and Urodynamics. 1995;14(1):57-64.

178. Tedroff K, Löwing K, Åström E. A prospective cohort study investigating gross motor function, pain, and health-related quality of life 17 years after selective dorsal rhizotomy in cerebral palsy. Developmental Medicine and Child Neurology. 2015;57(5):484-90.

179. Tedroff K, Löwing K, Jacobson DN, Aström E. Does loss of spasticity matter? A 10-year follow-up after selective dorsal rhizotomy in cerebral palsy. Developmental Medicine & Child Neurology. 2011;53(8):724-9.

180. Thomas SS, Buckon CE, Piatt JH, Aiona MD, Sussman MD. A 2-year follow-up of outcomes following orthopedic surgery or selective dorsal rhizotomy in children with spastic diplegia. Journal of Pediatric Orthopaedics-Part B. 2004;13(6):358-66.

181. Thomas SS, Aiona MD, Buckon CE, Piatt Jr JH. Does gait continue to improve 2 years after selective dorsal rhizotomy? Journal of Pediatric Orthopaedics. 1997;17(3):387-91.

182. Thomas SS, Aiona MD, Pierce R, Piatt Ii JH. Gait changes in children with spastic diplegia after selective dorsal rhizotomy. Journal of Pediatric Orthopaedics. 1996;16(6):747-52.

183. Tichy M, Kraus J, Horinek D, Vaculik M. Selective posterior rhizotomy in the treatment of cerebral palsy, first experience in Czech Republic. Bratislavské lekárske listy. 2003;104(2):54-8.

184. Touyama M, Touyama J, Aguni A. Postoperative course of a patient undergoing selective dorsal rhizotomy for cerebral palsy. No To Hattatsu. 2011;43(4):277

185. Trost JP, Schwartz MH, Krach LE, Dunn ME, Novacheck TF. Comprehensive short-term outcome assessment of selective dorsal rhizotomoy. Developmental Medicine & Child Neurology. 2008;50(10):765-71.

186. Turi M, Kalen V. The risk of spinal deformity after selective dorsal rhizotomy. Journal of Pediatric Orthopaedics. 2000;20(1):104-7.

187. Van Campenhout A, Huenaerts C, Poulussen L, Prinsen SD, Desloovere K. Role of femoral derotation on gait after selective dorsal rhizotomy in children with spastic cerebral palsy. Developmental Medicine and Child Neurology. 2019;61(10):1196-+.

188. VandeWiele BM, Staudt LA, Rubinstien EH, Nuwer M, Peacock WJ. Perioperative complications in children undergoing selective posterior rhizotomy: A review of 105 cases. Paediatric Anaesthesia. 1996;6(6):479-86.

189. van Schie PEM, Schothorst M, Dallmeijer AJ, Vermeulen RJ, van Ouwerkerk WJR, Strijers RLM, et al. Short- and long-term effects of selective dorsal rhizotomy on gross motor function in ambulatory children with spastic diplegia Clinical article. Journal of Neurosurgery-Pediatrics. 2011;7(5):557-62.

190. van Schie PEM, Vermeulen RJ, van Ouwerkerk WJR, Kwakkel G, Becher JG. Selective dorsal rhizotomy in cerebral palsy to improve functional abilities: evaluation of criteria for selection. Childs Nervous System. 2005;21(6):451-7.

191. Veerbeek BE, Lamberts RP, Kosel E, Fieggen AG, Langerak NG. More than 25 years after selective dorsal rhizotomy: physical status, quality of life, and levels of anxiety and depression in adults with cerebral palsy. Journal of Neurosurgery. 2022;136(3):689-98.

192. Veerbeek BE, Lamberts RP, Fieggen AG, Verkoeijen PPJL, Langerak NG. Daily activities, participation, satisfaction, and functional mobility of adults with cerebral palsy more than 25 years after selective dorsal rhizotomy: a long-term follow-up during adulthood. Disability and Rehabilitation. 2021;43(15):2191-9.

193. Veerbeek BE, Lamberts RP, Fieggen AG, Mankahla N, de Villiers RVP, Botha E, et al. A long-term follow-up study of spinal abnormalities and pain in adults with cerebral palsy and spastic diplegia more than 25 years after selective dorsal rhizotomy. Journal of neurosurgery Spine. 2020;34(2):228-35.

194. Wach J, Yildiz ÖC, Sarikaya-Seiwert S, Vatter H, Haberl H. Predictors of postoperative complications after selective dorsal rhizotomy. Acta Neurochirurgica. 2021;163(2):463-74.

195. Wang B, Zhang X, Fang XT. Limited selective posterior rhizotomy combined with adductor tenotomy for the improvement of motor ability of children with spastic lower limbs in cerebral palsy. Chinese Journal of Clinical Rehabilitation. 2005;9(19):218-20.

196. Wang DH, Li XZ, Ma L, Zhang X. Value of comprehensive rehabilitation intervention on functional recovery of children with spastic cerebral palsy following selective posterior rhizotomy. Chinese Journal of Clinical Rehabilitation. 2006;10(4):57

197. Wang R, Jiang W, Wei M, Wang J, Yu X, Xiao B, et al. Short-term change of tibial torsion in children with spastic cerebral palsy after selective dorsal rhizotomy. Translational Pediatrics. 2023;12(12):2131-41.

198. Wang Q, Hou T, Zhang C, Nian S, Wu Y, Yang X, et al. Long-term outcome of selective posterior rhizotomy for spastic cerebral palsy. Zhonghua wai ke za zhi [Chinese journal of surgery]. 1998;36(11):674

199. Wei M, Jiang WB, Zhan QJ, Li S, Liu C, Xiao B. Efficacy and influencing factors of selective dorsal rhizotomy for the treatment of spastic cerebral palsy in children. Chinese Journal of Contemporary Neurology and Neurosurgery. 2023;23(5):405-11.

200. Westbom L, Lundkvist Josenby A, Wagner P, Nordmark E, Westbom L, Lundkvist Josenby A, et al. Growth in children with cerebral palsy during five years after selective dorsal rhizotomy: a practice-based study. BMC Neurology. 2010;10:57-.

201. Wong AMK, Chen CL, Hong WH, Tang FT, Lui TN, Chou SW. Motor control assessment for rhizotomy in cerebral palsy. American Journal of Physical Medicine & Rehabilitation. 2000;79(5):441-50.

202. Wong AMK, Pei YC, Lui TN, Chen CL, Wang CM, Chung CY. Comparison between botulinum toxin type A injection and selective posterior rhizotomy in improving gait performance in children with cerebral palsy. Journal of Neurosurgery. 2005;102(4):385-9.

203. Wright FV, Sheil EM, Drake JM, Wedge JH, Naumann S. Evaluation of selective dorsal rhizotomy for the reduction of spasticity in cerebral palsy: a randomized controlled tria. Developmental medicine and child neurology. 1998;40(4):239‐47.

204. Xu J, Xu L, Zeng J, Yang XK, Li ZH, Shao GK, et al. Clinical observation of selective posterior rhizotomy for improving spasticity and gross movement in patients with cerebral palsy. Zhongguo gu shang = China journal of orthopaedics and traumatology. 2019;32(9):815

205. Xu L, Hong Y, Wang AQ, Wang ZX, Tang T. Hyperselective posterior rhizotomy in treatment of spasticity of paralytic limbs. Chinese medical journal. 1993;106(9):671-3.

206. Xu L, Li S, Gong H. Effect of selective posterior rhizotomy on children with spastic cerebral palsy. Chinese Journal of Rehabilitation Theory and Practice. 2003;9(10):629-30.

207. Yang TF, Chan RC, Wong TT, Bair WN, Kao CC, Chuang TY, et al. Quantitative measurement of improvement in sitting balance in children with spastic cerebral palsy after selective posterior rhizotomy. American Journal of Physical Medicine & Rehabilitation. 1996;75(5):348-52.

208. Yi B, Xu L, Hong Y. Lumbar structural observation of children with cerebral palsy after selective posterior rhizotomy. Zhonghua yi xue za zhi. 2001;81(16):983

209. Zaino NL, Steele KM, Donelan JM, Schwartz MH. Energy consumption does not change after selective dorsal rhizotomy in children with spastic cerebral palsy. Developmental Medicine and Child Neurology. 2020;62(9):1047-53.

210. Zhan QJ, Yu XD, Jiang WB, Shen M, Jiang SY, Mei R, et al. Whether the newly modified rhizotomy protocol is applicable to guide single-level approach SDR to treat spastic quadriplegia and diplegia in pediatric patients with cerebral palsy? Childs Nervous System. 2020;36(9):1935-43.

211. Zhang ZY, Zhang X, Yang YX, Zhang YF. Amelioration of the ability of independent ambulation of children with spastic cerebral palsy by limited selective posterior rhizotomy plus limited selective soft tissue release: A nine-year follow-up in 438 cases. Chinese Journal of Clinical Rehabilitation. 2005;9(47):105

212. Zheng JK, Chen GZ, Lin XC. Microscopically selective posterior rhizotomy for the treatment of cerebral palsy in children. Chinese Journal of Clinical Rehabilitation. 2002;6(4):489

213. Zhou N, Che S, Zhou B, Deng Z, Zhang J, Yang X. Effects of selective posterior rhizotomy on spastic cerebral palsy. Chinese Journal of Clinical Rehabilitation. 2003;7(2):348-9.

214. Zhou N, Chen SL, Zhou B, Deng ZQ, Tang WY. Retrospective study on selective posterior rhizotomy for treatment of spastic cerebral palsy in 85 cases. Chinese Journal of Clinical Rehabilitation. 2005;9(23):201
