# Supplementary figures and images for: Outcomes of selective dorsal rhizotomy in ambulatory children and young people with cerebral palsy: A scoping review
Source: Dev Med Child Neurol. 2025 Sep 19;68(2):175–86. doi: 10.1111/dmcn.16496 (PMC12766555; doi:10.1111/dmcn.16496)

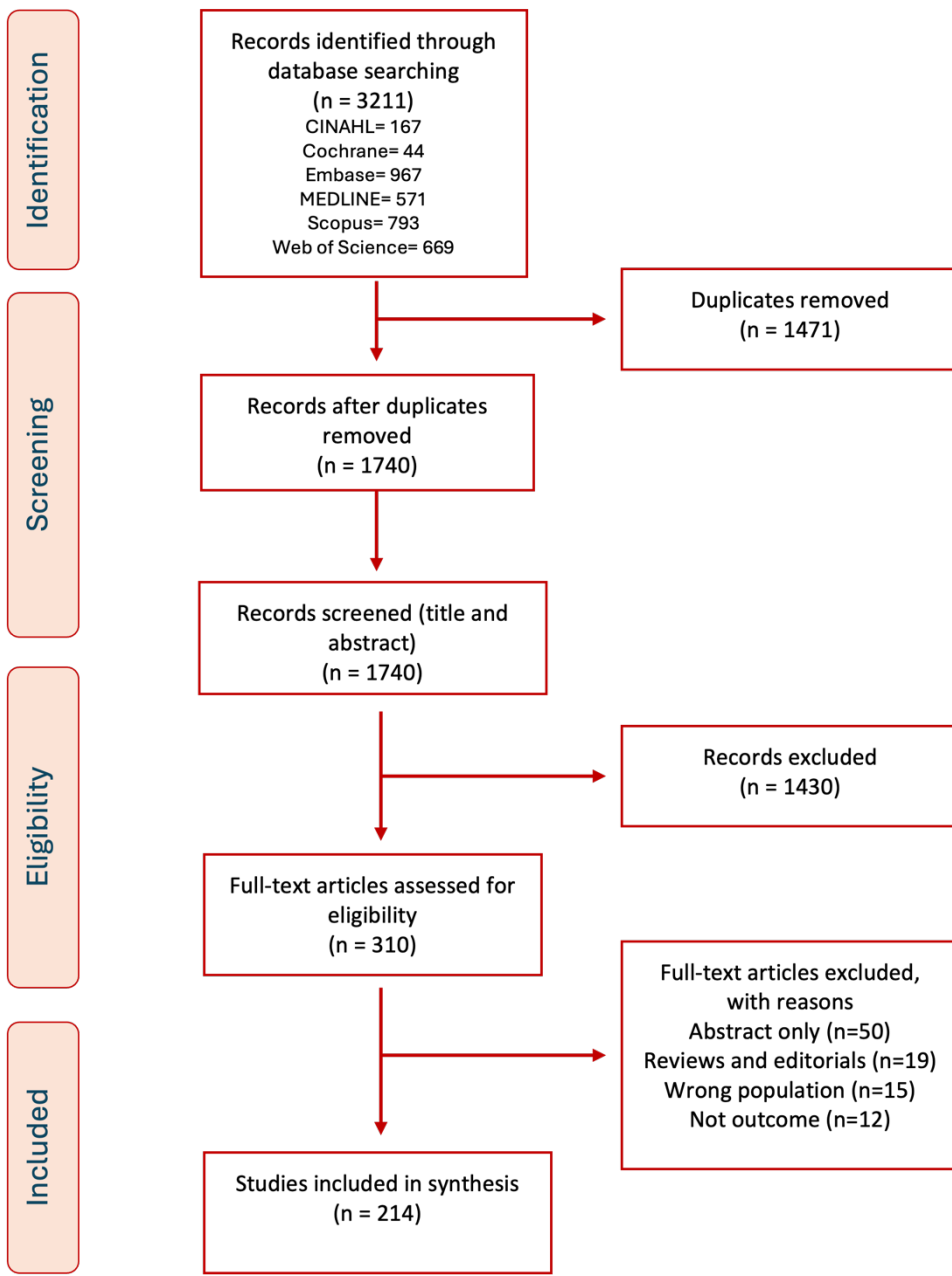

Supplement: Supplementary file 7 — Figure S1: PRISMA flow diagram. [file DMCN-68-175-s007.pdf]
